# The building concept of border defence facilities of Qin: Watchtowers along the King of Zhaoxiang great wall in Shaanxi province

**DOI:** 10.1371/journal.pone.0329298

**Published:** 2025-08-26

**Authors:** Xingyi Li, Yangyang Tong, Xiaoning Ma, Jiachen Chang, Binyan He

**Affiliations:** School of Cultural Heritage, Northwest University, Xi’an, China; Shenyang Jianzhu University, CHINA

## Abstract

Qin was a military power during the Warring States period, and in its confrontations with other regimes, it constructed a series of border defense projects, among which the most iconic is the Great Wall. Watchtowers are an important part of the Great Wall defense system, and their function was to provide soldiers with a better vantage point and shooting position. This paper studies the 347 watchtowers along the King of ZhaoXiang Great Wall (ZXGW) in Shaanxi Province, categorizes their architectural forms, and determines the distribution characteristics, and aims to understand the construction philosophy of the Qin state’s border defense projects through the early prototypes of watchtowers. These watchtowers were built by earth on-site, and likely with subsidiary buildings. Relevant literature and analysis on ArcGIS show different distribution characteristics and defense capability across the different geographical environments in the east, middle, and west sections. The watchtowers of the western section, situated in the Baiyu mountainous area, are taller and most densely distributed, thus offering the most significant defensive advantage of the three sections. The watchtowers in the eastern section, located in a relatively flat and hilly area with the lowest heights and distributed sparsely, provide the weakest defensive capability. The middle section serves as a transition zone between the east and west sections, with the height, distribution density, and defense advantages of watchtowers occupying a similar middle-ground position.

## 1. Introduction

### 1.1. The ZXGW

The Qin state was a military powerhouse during the Warring States period, defeating other regimes and ultimately unifying China. King Zhaoxiang was the monarch of the Qin state in the late Warring States period. During his 55-year reign as king, he continuously expanded the territory, achieving numerous victories in wars. After winning the war against the Yiqu Rong people in the north, King Zhaoxiang built the Great Wall, which is approximately 800 kilometers [1], and is thus also referred to as the ZhaoXiang Great Wall(ZXGW) [2].The ZXGW is extensive, traversing various terrains along its route, and different sections reflect different construction principles [3,4], further revealing the defensive strategies of the Qin state at its borders. To address this concern, an exhaustive survey of the ZXGW in Shaanxi Province was conducted, yielding a substantial collection of detailed and specific graphic and textual materials. These materials encompass representations of the wall, watchtowers, beacon towers, and forts.

Based on the Historical records, it can be inferred that, the construction of the King of The ZXGW dates back to the Warring States period and was in use from the Warring States period to the early Han Dynasty [5]. In 272 BC, Empress Dowager Xuan deceived and killed the King of the Yiqu Rong at Ganquan, and then raised an army to attack the remnants of the Yiqu. As a result, Qin controlled Longxi, Beidi, and Shangjun (the northern region of Shaanxi north of the Ching River), and built the ZXGWto defend against the Hu. Initially, the purpose of the ZXGW was to safeguard the newly acquired Yiqu territory and demarcate the Qin state’s national boundary. Subsequently, with the expansion of the northwest territory of Qin State, when Emperor Qin Shihuang occupied the land south of the Yellow River (modern-day Hetao Plain), the ZXGW was no longer positioned on the empire’s boundaries and lost its strategic significance.

**Fig 1:**
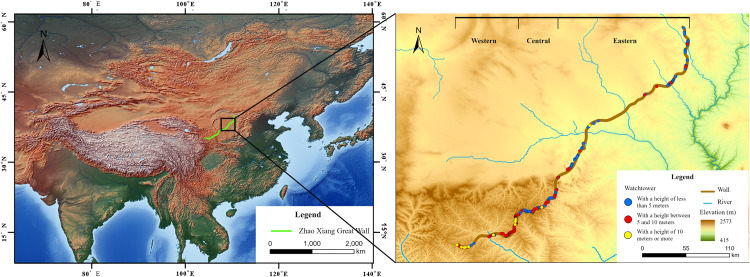
Distribution of the ZXGW in Shaanxi province. (a)The base data was from Nature earth: file:///D:/Users/14733/Downloads/HYP_HR_SR_OB_DR/HYP_HR_SR_OB_DR.README.html. The green line shows the distribution of the ZXGW. (b)The base data was from Earth Explore: https://earthexplorer.usgs.gov/. The brown line refers to the distribution of ZXGW in Shaanxi Province. The blue line shows the distribution of rivers in the region. The blue dots refer to watchtowers below 5 metres; the red dots refer to watchtowers between 5 and 10 metres; and the yellow dots refer to watchtowers above 10 metres. The darker the brown colour on the map, the higher the altitude, and the darker the green colour, the lower the altitude.

The ZXGW crawls across the Inner Mongolia Autonomous Region, the Shaanxi, Ningxia, and Gansu provinces [6]. In particular, the Shaanxi section of the Great Wall extends into Shenmu City, Yuyang and Hengshan Districts, Jingbian County of Yulin City, as well as Zhidan and Wuqi Counties of Yan’an City [2], and can be divided into three segments (Fig 1). The eastern section (from the watchtower in Yangwangta village to the watchtower in Lunzehao village) extends between Maosu desert to the north and the Loess Plateau gullies in the south, a landscape mostly comprising low hills and low altitude. The central (from watchtower No.1 in Qiaojiawaze village to the watchtower in Laofenyaoxian village) and western sections (from the watchtower in Hejiagou village to the watchtower No.5 in Lingouliang village) situated in mountainous areas on the north and south slopes of the Baiyu Mountain Range, respectively, across higher and more undulating terrain than the eastern side. In the western section, in particular, the characteristics of the Loess Plateau are more prominent, with crisscrossing gullies and alternating distribution of mountains and valleys.

Geographically, the Qin Great Wall constructed in Shaanxi Province during King Zhaoxiang’s reign (306–251 BC) was situated along the northern boundary of the Guanzhong Plain. Notably, this segment was proximate to the Qin capital of Xianyang—approximately 400 km—compared to the sections in Gansu and Inner Mongolia, thereby constituting the primary defensive line safeguarding the political hub of Xianyang. The persistent threat from the Rong tribe has led to particularly intense military conflicts in northern Shaanxi. This is corroborated by recent archaeological discoveries at sites such as Zhaitouhe Cemetery [7], Shijiahe Cemetery [8], and Wohuwan Warring States Tomb [9], which have produced a rich array of cultural artifacts linked to the Rong people. These discoveries verify that this region served as the principal hub of Rong tribal activity during the Warring States period. Moreover, a significant number of arrowheads and spearheads from the late Warring States and Qin periods have been excavated at sites in proximity to Shenmu and Yulin [10]. In addition, the discovery of carbonized wood remnants and signaling fire traces at beacon tower sites along the wall [11] offers tangible evidence of the frequent state of alert and conflict that characterized this region.

Historical records indicate frequent military interactions along the Shaanxi section of the Warring States Qin Great Wall. In 318 B.C., Yiqu launched a surprise attack on Qin from the rear while its military was engaged with the League of Five States [12]. Following the assassination of the Yiqu king in 272 B.C. at Ganquan Palace, King Zhaoxiang initiated the construction of the Great Wall to safeguard against the residual Yiqu forces, underscoring the strategic significance of this frontier [13]. During the late Warring States era, sporadic raids by the nomadic Xiongnu and the Linhu and Loufan tribes from the Ordos region heightened the defensive burden on this segment of the wall [14]. The strategic geographical location of Shaanxi, coupled with the military threats it faced, rendered the Qin Great Wall’s Shaanxi section especially significant for analyzing the Qin’s frontier defense strategy during this pivotal juncture preceding imperial unification.

### 1.2. The watchtowers

Over the centuries of construction, the Great Wall encompassed various architectural elements such as walls, watchtowers, buttresses, beacon towers, passes, and forts [15]. The composition of the ZXGW in Shaanxi Province (426 km of wall, 347 watchtowers, 14 buttresses, 89 beacon towers, 14 passes, and 8 forts) highlights the efforts and sophistication put in planning this section, which became the prototype of the later Great Wall [2].

Watchtowers are a common subsidiary facility across the Great Wall, but their definition has remained complex since ancient times. Before the Ming Dynasty, it mostly referred to defensive facilities on walls of city [16–18], yet by the Chenghua period of the Ming Dynasty, the term began to refer to defensive facilities of the Great Wall defense system [19]. According to historical records, some were built on top of walls, while others were incorporated within a wall or stood outside it [20]. This paper adopts the definition of watchtower provided in the *Resources’ Investigation Report of the Early Great Wall in Shaanxi Province*, wherein a watchtower is characterized as a high platform protruding from the Great Wall [2].

The construction and usage time of the watchtowers along ZXGW is approximately the same as the ZXGW. Almost all of the watchtowers are surrounded by tiles decorated with dot marks, and a significant number are encircled by tiles decorated with textile marks. The former are specific representatives of tiles of the Qin state and Qin Dynasty, having been discovered at various sites associated with the Qin people, such as the Qin Yong City Sites and the Mausoleum of the First Qin Emperor. The latter are characteristic of Han Dynasty tiles, found in large quantities at the Han Capital, Chang’an, and the Western Han Dynasty imperial Mausoleums [2]. This evidence indicates that these watchtowers were originally constructed by the Qin people, and later were repaired and utilized by the Han people.

### 1.3. Literature review and main issues

Hitherto, research on watchtowers has mainly focused on architectural features and confirms that the watchtowers in the early stage were solid rammed earth platforms [21–23], and that the brick-covered hollow watchtower type did not appear until the Ming Dynasty [3,24,25]. The hollow watchtowers exhibit a diverse range of forms, with some papers conducting typological analysis based on building materials and spatial structures as classification criteria [26–32]. In terms of location, watchtowers are always built on high ground to offer a broader overview of the surrounding area [33]. At the same time, they are often found on mountain slopes facing outward so that soldiers could effectively repel attacks when advancing troops were at a disadvantage, moving slowly uphill [2]. Moreover, to observe enemy movement effectively and defend against attacks, the layout of the watchtowers took into account the requirement for visibility between neighboring ones [34] and the range of missile weapons to create lethal firing zones against invading forces between watchtowers [35]. As far as functionality is concerned, watchtowers could provide good observation and shooting positions, accommodate troops, and store combat materials [36,37]. It has also been argued that watchtowers functioned as facilitators of military intelligence by disseminating information via beacon towers [38], thus forming an information transmission network between watchtowers, beacon towers, and forts along the Great Wall [39–41]. Some papers combine historical documents, stone carvings along the Great Wall, and stratigraphy to examine the marking [42] and classification of the watchtowers in the Ming dynasty [43,44], personnel and material management [45,46], material allocation [47,48], and construction projects [49].

Overall, most studies on watchtowers focus on the Ming Great Wall, and the studies on the pre-Ming Dynasty part are relatively weaker. Focusing only on the Ming watchtowers may hinder a comprehensive understanding of their emergence and evolution, and is not conducive to a systematic evaluation and understanding of the Great Wall’s defense system.

### 1.4. Research aims

The primary objective of this study is to elucidate the guiding principles behind Qin’s military strategy in constructing border defenses, focusing on the watchtowers along the ZXGW during the Warring States period. This research specifically investigates the 347 watchtowers situated along the ZXGW in Shaanxi Province. It delves into the systematic patterns observed in the watchtowers, considering aspects such as plan shape, size, and spacing. Furthermore, the study evaluates their architectural characteristics and underlying construction principles. By capturing these features, it provides insights into the nascent stages of the Great Wall’s development. These findings are then contextualized within the historical backdrop to offer a comprehensive understanding of the large-scale border defense mechanisms employed by the Qin State.

## 2. Methodology

MySQL was used to record the information on 347 watchtowers collected in the survey; a database of watchtowers along the early Great Wall in Shaanxi Province was created, with each watchtower as a separate document in the database with several fields, such as construction materials, construction methods, plane shapes, and dimensions. Subsequently, ArcGIS was used to visualize and statistically analyze the objects in the database, to overlay the watchtower’s coordinate information with topography, river, slope and precipitation data, and to estimate the density through the watchtower’s coordinates, so as to investigate the purpose behind the construction of the watchtowers and the principles of their layout from the perspective of physical geography characteristics and coordination between the defense facilities. And linear regression analysis is employed to rigorously examine the complex interrelationships among the research variables, thereby establishing statistically significant correlations between the investigated factors. Finally, by integrating the data and visual analyses discussed earlier, the paper provides an overview of the architectural and distributional characteristics of these watchtowers, as well as insights into the reasons for these features in terms of the natural environment and historical documents.

### 2.1. Kernel density

**Fig 2:**
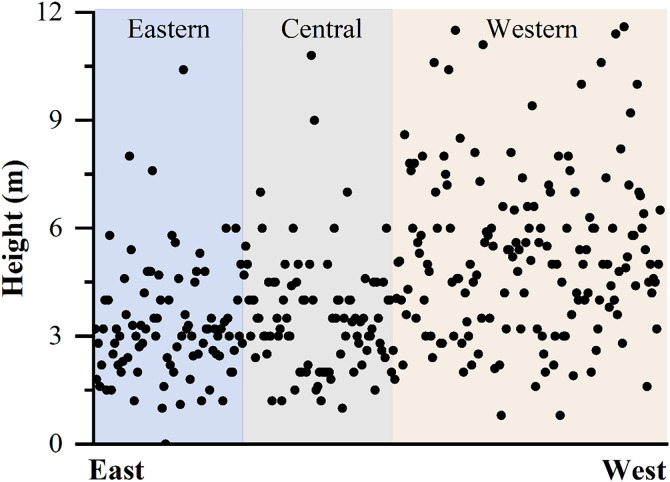
Height distribution of watchtowers along the ZXGW in Shaanxi province, oriented from East to West. The horizontal axis corresponds to the relative east-west positional sequence of these watchtowers, while the vertical axis indicates their height measurement. The light blue section represents the eastern portion of the Shaanxi segment of the ZXGW, the light gray section signifies the central portion, and the salmon pink section denotes the western portion. Black dots are used to represent watchtowers that are spread along the Great Wall.

Kernel density estimation is a technique to estimate the probability density function [50]. Based on the quartic kernel function described in Silverman [51], ArcGIS Pro created a Kernel Density tool to calculate the density of features. The principle of this analysis tool assumes a smoothly curved surface fitted over each point. The surface value is highest at the location of the point and diminishes with increasing distance from the point, reaching zero at the search radius distance from the point. In this paper, kernel density analysis was used to generate two figures (Figs 1 and 2), which calculated the kernel density of the existing watchtowers and the kernel density of the watchtowers after adding recovery points respectively.

The predicted density at a new (x, y) location is determined by the following formula:


$$\begin{array}{c} Density=\frac{\text{1}}{(radius{)}^{\text{2}}}\sum_{i=\text{1}}^{n}\left[\frac{3}{\pi }*{\left(\text{1}-{\left(\frac{{dist}_{i}}{radius}\right)}^{\text{2}}\right)}^{\text{2}}\right] \end{array}$$ (1)

$$For\backslash {dist}_{i}<\;radius$$

where:

*radius refers to the search radius.*

*i = 1,…,n represents the i-th watchtower point within the search radius of the (x,y) location.*

${dist}_{i}$ is the distance between each watchtower and the (x,y) location.

This formula first calculates the squared normalized distance, converting the distance into a ratio within the range of [0, 1).$\text{1}-{\left(\frac{{dist}_{i}}{radius}\right)}^{\text{2}}$serves as a weight decay function, which assigns greater weights to watchtowers closer to the point (x, y) and smaller weights to those farther away. Next, ${\left(\text{1}-{\left(\frac{{dist}_{i}}{radius}\right)}^{\text{2}}\right)}^{\text{2}}$ accelerates the decay rate of weights for distant watchtowers. It emphasizes the contribution of nearby watchtowers to the density value and weakens the influence of distant ones. This approach prevents the density value from suddenly dropping to zero at the edge of the search radius, contributing to a smoother image. Then, by multiplying by the normalization coefficient $\frac{3}{\pi }$, it ensures that the total integral of the kernel function within the search radius is 1, satisfying the property of probability density. This guarantees the scientific validity and comparability of the density values when the search radius changes before and after adding the recovery points. After that, the calculation results of all watchtowers within the search radius are summed up. Finally, by multiplying by $\frac{\text{1}}{(radius{)}^{\text{2}}}$, the total weight is standardized according to the search area, ensuring that the density value represents the contribution of watchtowers per unit area rather than an absolute cumulative value.

The algorithm used to determine the default search radius, also known as the bandwidth, is as follows:


$$SearchRadius=\text{0}.\text{9}*min\left(SD\text{,}\sqrt{\frac{\text{1}}{ln(\text{2})}}*D_{m}\right)*n^{-\text{0}.\text{2}}$$ (2)

where:

$\;\;\;\;\;\;\;\;D_{m}$ is the median distance from the mean center.

n is the total number of watchtowers.

        SD is the standard distance, and its calculation formula is as follows.


$$SD=\sqrt{\frac{\sum_{i=\text{1}}^{n}{\left({\text{x}}_{\text{i}}-\overset{―}{\text{X}}\right)}^{\text{2}}}{n}+\frac{\sum_{i=\text{1}}^{n}{\left({\text{y}}_{\text{i}}-\overset{―}{\text{Y}}\right)}^{\text{2}}}{n}+\frac{\sum_{i=\text{1}}^{n}{\left({\text{z}}_{\text{i}}-\overset{―}{\text{Z}}\right)}^{\text{2}}}{n}}$$ (3)

where:

${\text{x}}_{\text{i}}$, ${\text{y}}_{\text{i}}$ and ${\text{z}}_{\text{i}}$ are the coordinates for each watchtower.

{$\overset{―}{\text{X}}$, $\overset{―}{\text{Y}}$, $\overset{―}{\text{Z}}$} represents the mean center for the features.

n is the total number of watchtowers.

The search radius for kernel density needs to be able to reflect both the overall distribution characteristics of the watchtowers and capture local detailed features. This formula compares the standard distance (SD) and the median distance ($D_{m}$) of the watchtowers. Multiplying $D_{m}$ by$\sqrt{\frac{\text{1}}{ln(\text{2})}}$ is to convert$D_{m}$ to a scale that matches SD, ensuring comparability between the two. SD is sensitive to a few isolated watchtowers far from the core area, while $D_{m}$ is not. Selecting the minimum value of the two helps to choose a reasonable radius. $n^{-\text{0}.\text{2}}$ is used to adjust the search radius. When no restoration points are added, the total number of watchtowers is small, and the calculated search radius is large. This helps to capture the distribution characteristics of the density of watchtowers over a larger area. When recovery points are added, the total number of watchtowers is large, and the calculated search radius is small. A smaller search radius helps to capture more local features. Finally, multiply the result by 0.9. 0.9 is an empirical coefficient. Slightly reducing the search radius helps to make the results more conservative and robust [52].

### 2.2. Linear regression

In examining the interrelations among research variables, this study utilizes linear regression analysis to ascertain the existence of genuine correlations between factors. IBM SPSS Statistics 25.0 was used to calculate regression coefficients, significance levels, and model fit, as well as to test the assumptions of residual normality and homoscedasticity. The significance threshold was set at α = 0.05 with a two-tailed test. To explore the relationship between watchtower height and section (a categorical variable, coded as Eastern = 1, Central = 2, Western = 3), the model takes watchtower height as the dependent variable and section as the independent variable. For investigating the relationship between the height and the planar shape of watchtowers (a categorical variable, coded as Type A = 1, Type B = 2, Type C = 3), the model uses watchtower height as the dependent variable and planar shape as the independent variable. When examining the relationship between the kernel density value of the watchtower location and section (a categorical variable, coded as Eastern = 1, Central = 2, Western = 3), the model regards the kernel density value as the dependent variable and section as the independent variable.

Linear regression analysis is a statistical technique used to model the relationship between a dependent variable and one or more independent variables. Based on the ordinary least squares method described in statistical literature, this study employed linear regression to analyze the relationships among variables. The principle of this analysis method assumes a linear relationship between the dependent variable and the independent variable(s), with the goal of finding the best-fitting straight line through the data points. The line’s position is determined by minimizing the sum of squared differences between the observed values and the values predicted by the linear model. In this paper, regression analysis was used to examine three relationships: (1) between watchtower height and section (2) between the height and planar shape of watchtowers, and (3) between kernel density values and planar shape classification.

The predicted value of the dependent variable Y at a given value of X is determined by the following formula:


$$\;\text{Y}=\beta_{\text{0}}+BX+\varepsilon \mathrm{\backslash ]}$$ (4)

where:

Y is the Dependent Variable. In the regression analysis of this paper, the dependent variables are the height of the watchtower and the kernel density value respectively.

X is the Independent Variable. In the regression analysis of this paper, the independent variables are regional segmentation (Eastern = 1, Central = 2, Western = 3) and planar shape classification (Type A = 1, Type B = 2, Type C = 3) respectively.

$\beta_{\text{0}}$: Intercept.

∊ is the Error Term, representing the random variation unexplained by the model.

B is the Regression Coefficient, which represents the average change in Y when X increases by 1 unit.


$$B=\frac{\sum_{i=\text{1}}^{n}\left(X_{i}-\overset{―}{X}\right)\left(Y_{i}-\overset{―}{Y}\right)}{\sum_{i=\text{1}}^{n}{\left(X_{i}-\overset{―}{X}\right)}^{\text{2}}}$$ (5)

where:

${\text{X}}_{\text{i}}$ and ${\text{Y}}_{\text{i}}$ are the observed values of the independent and dependent variables for the i-th watchtower.

$\overset{―}{\text{X}}$ and $\overset{―}{\text{Y}}$ are the mean values of the independent and dependent variables respectively.

n denotes the number of watchtowers. In this study, the number of watchtowers is 347 in the regression analysis between the kernel density of watchtower locations and the “section” variable. In the regression analysis related to watchtower height, the number is 344 because the heights of 3 watchtowers are unmeasurable due to severe collapse.

Formula (5) first calculates the covariance between X and Y, measuring how much the two variables change together. The denominator calculates the variance of X, measuring how much X varies on its own. Dividing the covariance by the variance gives the slope of the regression line, which represents how much Y changes on average for a unit change in X. This approach gives more weight to data points that are farther from the mean of X, as these points provide more information about the relationship between the variables.

A t-test was employed to assess the statistical significance of regression coefficients (P), determining whether the independent variable (X) significantly affects the dependent variable (Y). The test statistic is calculated as follows:


$$t=\frac{B}{SE(B)}\;\text{,}\;\text{Degrees}\;\text{of}\;\text{freedom}:\;\text{df}=\text{n}-\text{2}$$ (6)

where:

The linear regression model needs to estimate two unknown parameters: the intercept $\beta_{\text{0}}$ and the slope B. According to the definition of degrees of freedom, the degrees of freedom are equal to the sample size minus the number of parameters to be estimated. Therefore, in this paper, the degrees of freedom for the t-test is df = n − 2, which reflects the constraints of the model on the data and the remaining information.

SE(B) is the standard error of B, reflecting estimation uncertainty.


$$SE(B)=\sqrt{\frac{\sum_{i=\text{1}}^{n}{\left(Y_{i}-\hat{Y_{i}}\right)}^{\text{2}}}{(n-\text{2})\cdot \sum_{i=\text{1}}^{n}{\left(X_{i}-\overset{―}{X}\right)}^{\text{2}}}}$$ (7)

Formula (7) first calculates, namely the Sum of Squared Residuals, which represents the sum of the squares of the differences between the actual observed values and the model predicted values. It is used to measure the degree of insufficient fit of the model as a whole. Then, the Mean Squared Error is obtained by dividing the Sum of Squared Residuals by the degrees of freedom, so as to adjust the average level of the Sum of Squared Residuals. Finally, divide by the sum of squared deviations of the independent variable X from its mean and take the square root to obtain the magnitude of the sampling error of the regression coefficient B, the average fluctuation of the B values estimated from different samples.

t is obtained by dividing B by SE(B). If the absolute value of t is very large, it indicates that B is “sufficiently large” relative to its sampling fluctuation; if the absolute value of t is very small, it indicates that B may only be caused by sampling fluctuation and may actually be 0. Then, the p-value can be calculated from the t-statistic using SPSS. If p < 0.05, it indicates that B is significantly different from zero, meaning the effect of X on Y is statistically significant.

${\text{R}}^{\text{2}}$ is Coefficient of Determination, Measuring the proportion of the variance in the dependent variable (Y) that is predictable from the independent variable (X). Calculation formula:


$$R^{\text{2}}=\text{1}-\frac{\sum_{i=\text{1}}^{n}{\left(Y_{i}-\hat{Y_{i}}\right)}^{\text{2}}}{\sum_{i=\text{1}}^{n}{\left(Y_{i}-\overset{―}{Y}\right)}^{\text{2}}}$$ (8)

$\sum_{\text{i}=\text{1}}^{\text{n}}{\left({\text{Y}}_{\text{i}}-\hat{{\text{Y}}_{\text{i}}}\right)}^{\text{2}}$ is the Sum of Squared Residuals, and $\sum_{\text{i}=\text{1}}^{\text{n}}{\left({\text{Y}}_{\text{i}}-\overset{―}{\text{Y}}\right)}^{\text{2}}$ is the Total Sum of Squares. Dividing the former by the latter gives the proportion of the variance in Y that is unexplained by the model, while ${\text{R}}^{\text{2}}$ is the proportion of the total variance in Y that is explained by the model. R^2^ ranges from [0, 1]. Values closer to 1 indicate better model fit.

According to the above calculation method, the following Linear regression coefficients and model fit for the associations between different are obtained (Table 1) [53,54].

**Table 1:** Linear regression coefficients and model fit for the associations between different variables.

Model Associated Variables	Dependent variable	Independent variable	B(SE)	P	R²
**Watchtower Height and Section**	Watchtower Height	Section	1.22 (0.20)	< 0.001	0.102
**Watchtower Height and Planar Shape Classification**	Watchtower Height	Planar Shape Classification	−0.58 (0.20)	0.003	0.025
**Kernel Density Value and Section**	Kernel Density Value	Section	507.431 (22.298)	<0.001	0.600

### 2.3. Viewshed analysis

The Visual Reachable Domain Analysis Diagram presented in this paper utilizes the Observer Point analysis tool within ArcGIS. This tool calculates the visible area from each watchtower taking into account terrain and refractivity coefficient. The Visual Reachable Domain Analysis Diagram features hillshade as the underneath layer, overlaid by the visibility analysis layer. The visibility analysis layer uses a stretched color ramp to represent the visibility of each location on the map. Transparent areas indicate that the location is not visible to individuals on the watchtowers, while yellow areas show visibility [55].

### 2.4. Calculation data

In order to exclude the natural and human-made factors to watchtower destruction that affect the results of analyzing the distribution density of watchtowers, this article turns to the concept of “recovery density”. Based on the positional relationship between watchtowers and walls of the Great Wall, Recovery Density = number of remaining watchtowers/ length of remaining wall; Current Density = number of remaining watchtowers/ total length of wall; Recovery Spacing = length of remaining wall/ number of surviving watchtowers. Accordingly, the distribution density of watchtowers in the three sections along the ZXGW in Shaanxi Province is shown in Table 2 below.

**Table 2:** Distribution Analysis of the Watchtowers along the ZXGW in Shaanxi Province.

Sections	Total wall(m)	Remaining wall(m)	Remaining watchtowers (pcs)	Recovery density (pcs/km)	Exsisting density (pcs/km)	Recovery spacing(m)
Eastern	288521.9	68214.3	91	13	3	749.61
Central	68731.0	27520.0	92	33	13	299.13
Western	105577.0	39362.0	164	42	16	240.01

This study systematically examines the concept of watchtower spacing along the ZXGW, motivated by substantial variations in recovery distances observed across three distinct sections. The research implemented a methodological approach involving the strategic placement of reconstructed watchtower locations within a GIS framework. These reconstructed points were positioned according to two key parameters: (1) the recovery distances for each section, and (2) the inferred alignment of the original wall structure.

It should be noted that historical records of the distribution of ZXGW during the Warring States period are only found in the Records of the Grand Historian: The Biography of the Xiongnu [56]. The conciseness of these records necessitates confirmation of its specific distribution and direction through archaeological investigations. Following numerous investigations and scholarly studies, a general consensus has been reached regarding the primary route of the ZXGW [57–60]. It is important to note that this inquiry was predicated on the recognition of the research findings of preceding scholars. Due to factors such as natural erosion and human destruction, approximately 127 kilometers of the ZXGW in Shaanxi province have vanished. Comprehensive surveys and studies reveal that the ZXGW’s peak was originally tiled, and thus, numerous Qin tiles in Warring States period remain scattered along its walls. Notably, sections with a higher concentration of these tiles tend to be in worse condition, whereas areas with fewer or no tiles are relatively well-preserved. Given this pattern, these tiles, characterized by the Qin architectural style often featuring rope patterns or plain surfaces externally and hemp spot patterns internally, have become a crucial indicator for determining the wall’s direction. Moreover, during explorations of certain vanished sections, layers of compacted soil were unearthed, offering additional context for ascertaining the wall’s direction. By integrating these archaeological insights, researchers can scientifically and objectively reconstruct the specific orientation of the ZXGW, affirming the direction of the vanished sections discussed in this paper as objectively accurate. Through systematic documentation and quantitative analysis of these points, the study developed a scientifically grounded reconstruction of the Great Wall’s initial configuration. This method provides a robust basis for comparative analysis of defensive patterns among watchtowers across the three sections, facilitating examination of both convergent and divergent characteristics in their functional relationships.

In addition, this study reconstructs the number of watchtowers along the no-longer-extant portions of the ZXGW in Shaanxi Province based on verified survey data. The lengths of remaining walls and their associated watchtowers have been systematically recorded. Notably, the watchtowers were constructed as integral components of the Great Wall, with no freestanding or isolated structures, meaning their distribution strictly corresponds to the main wall’s layout. Given that the ZXGW was built following the principle of “adapting to local conditions,” the study divides the Shaanxi section into three distinct geomorphic units—eastern, central, and western—and calculates the average spacing of watchtowers within each unit. This approach captures variations in watchtower density due to differing geographical conditions, ensuring methodologically sound and objective results. While the exact positioning of watchtowers in disappeared segments would ideally require archaeological excavation, the immense scale of the Great Wall makes full-scale exploration unfeasible. Thus, the following estimation methods are employed (1) Documentation of remaining segments: The lengths of remaining walls and their corresponding watchtower counts are recorded, allowing for the calculation of average watchtower spacing in each geomorphic unit. (2) Estimation for disappeared segments: By applying the average spacing observed in remaining sections to the lengths of disappeared segments, the approximate number of watchtowers in these areas is inferred. Although this method assumes uniform watchtower spacing and cannot pinpoint individual watchtower’s locations, it provides a scientifically grounded approximation of watchtower quantities and distribution densities. These estimates serve as reliable data for subsequent analyses of defensive capabilities across different segments of the Great Wall.

## 3. The architectural characteristics of watchtowers

**Fig 3:**
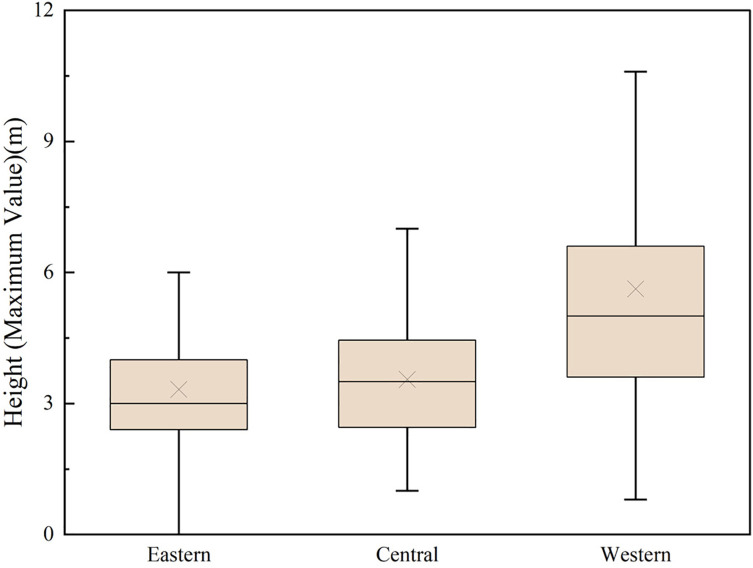
Height distribution of watchtowers along the ZXGW in Shaanxi Province in eastern, central, and western sectors. The figure’s box delineates the height distribution range of watchtowers across the eastern, central, and western sections of the ZXGW in Shaanxi Province. The horizontal coordinates indicate the different regions, and the vertical coordinates indicate the heights of the watchtowers. The whiskers above and below the box connect the maximum and minimum heights of the watchtowers, respectively. The upper end of the box, representing the 75th percentile, indicates that 75% of the data falls below this value. The middle horizontal line, showcasing the 50th percentile, serves as the median, bisecting the data into two equal parts. Conversely, the bottom horizontal line, typifying the 25th percentile, denotes that 25% of the data is situated below this mark. The central crosshair highlights the mean value of the dataset.

**Fig 4:**
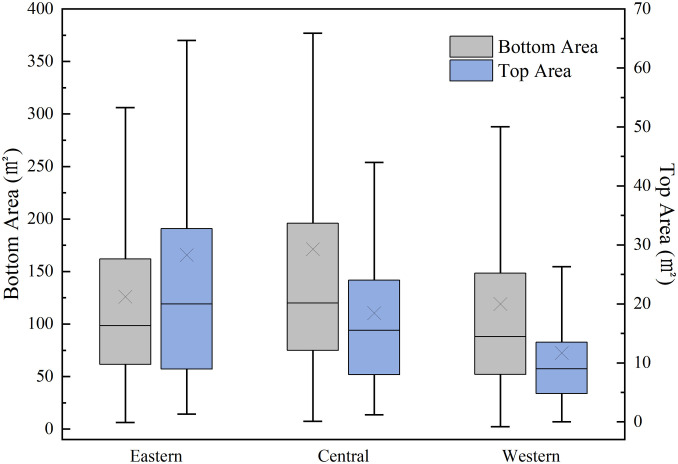
Distribution of summit and basal areas of watchtowers along the ZXGW in Shaanxi Province in eastern, central, and western sectors. The figure’s boxes symbolize the intervals between the distribution of area values at the base and apex of the watchtowers in the eastern, central, and western sections of the ZXGW in Shaanxi Province. The horizontal coordinates distinguish different areas, with the left vertical coordinates indicating the area at the base of the watchtower, and the right vertical coordinates representing the area at the top. The whiskers outside the box delineate the range of the area at either the top or bottom of the watchtower across various regions. The top horizontal line within the box signifies the data point at 75%, suggesting that 75% of the data is below this value. The middle horizontal line within the box denotes the 50% data point, implying that this is the median or center value of the data. The bottom horizontal line within the box indicates the 25% data point, meaning that 25% of the data falls below this value. Lastly, the fork in the middle of the box represents the average value of the data.

The remains of the watchtowers comprise mostly earthen mounds that protrude from one or both sides of the wall and rise above the wall. They were primarily rammed by locally sourced soil and are generally shaped as a conical or square frustum 2–12 meters high (Figs 2 and 3), narrower at the top (averaging 5.02m x 2.98m) and wider at their base (averaging 14.35m x 9.49m) (Fig 4). Almost all of the watchtowers are surrounded by massive tiles. This suggests that when the ancients utilized this section of the Great Wall, there were likely auxiliary buildings on or around the watchtower. The original height of the watchtower is challenging to determine due to millennia of rain erosion and wind ablation.

Determining the original height of the watchtowers presents significant challenges due to extensive weathering processes and artificial damage. Given the lack of complete preservation, this study adopts the baseline assumption that all watchtowers exhibit an equal probability of structural degradation. Firstly, it should be noted that the height data for the watchtowers currently in existence represent present-day measurements and there are no recorded histories of their initial construction heights available. Secondly, the sample size – comprising 347 watchtowers along the ZXGW in Shaanxi Province – is statistically representative. According to the law of large numbers, individual instances of damage to watchtowers, which might deviate from the mean, are not expected to significantly affect the overall average height. To ensure the robust validity of our research conclusions, we employed a stratified statistical method, carrying out independent analyses of the heights of watchtowers within different topographical units. The statistical results reveal significant disparities in the average heights of watchtowers across the three distinct topographical segments (3.32m in the eastern section, 3.55m in the central section, and 5.63m in the western section). This discovery provides quantifiable evidence to support the contention that the ZXGW implemented differentiated construction strategies in response to diverse topographical conditions.

Although the uniformity of loss rates cannot be directly verified, if the loss rates of watchtowers are assumed to be uneven, this could theoretically lead to certain diachronic differences in watchtower density and architectural characteristics across different sections. However, the regression analysis results suggest that such potential variations caused by uneven loss rates are not substantial enough to obscure or alter the inherent core differences in watchtowers among sections. Regarding the impact of loss rates on watchtower density across sections, linear regression analysis was conducted, yielding an R^2^ = 0.60. This indicates that the independent variable “section” explains 60.0% of the variance in the dependent variable “kernel density value of watchtower locations,” establishing it as the primary influencing factor. Consequently, the effect of uneven loss rates on watchtower density is subordinate to that of section. Even if loss rates are indeed uneven, the dominance of section ensures the validity of the conclusions drawn in this study regarding watchtower density. To assess the influence of loss rates on the architectural characteristics of watchtowers across sections, a linear regression analysis was performed with “section” as the independent variable and “watchtower height” as the dependent variable. The results show that section accounts for 10.2% of the variance in watchtower height, confirming that section is a contributing factor. Furthermore, the regression analysis yielded a statistically significant association (p < 0.001), indicating that the relationship between section and watchtower height is non-random.

The comparative map illustrates the absence of a consistent correlation between the size of watchtowers and the distinct geomorphic environments of the eastern, central, and western zones. However, the height of the watchtowers demonstrates a noticeable disparity along the ZXGW in Shaanxi Province. Specifically, the watchtowers located in the western section are significantly taller than those in the eastern section, while the watchtowers in the middle section exhibit a height that falls between the two extremes.

**Fig 5:**
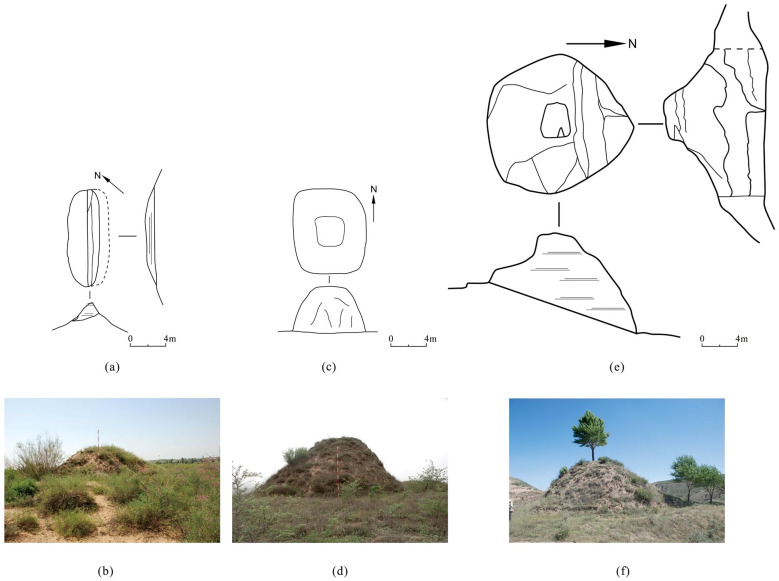
The Plan, profile and elevation images of watchtowers along the ZXGW in Shaanxi province of different heights and photographs of representative watchtowers. The base photos were taken by Xingyi Li. (a)and (b): Watchtower No.1 in Dongzuojie village (height of less than 5 meters). (c) and (d): Watchtower No. 2 in Zhongyangqing village (with a height between 5 and 10 meters. (e) and (f): Watchtower No. 1 in Dongjian village (with a height of more than 10 meters).

**Fig 6:**
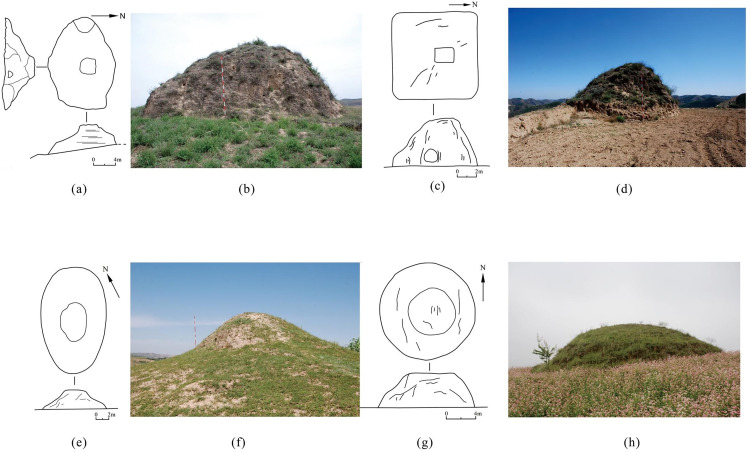
Plan, profile and elevation images of watchtowers along the ZXGW in Shaanxi province at different Planar Shapes and photographs of representative watchtowers. The base photos were taken by Xingyi Li. (a) and (b): The watchtower in Liugou village (irregular shape). (c) and (d): Watchtower No.2 in Malianyaoxian village (rectangular shape). (e) and (f): The watchtower in Liujian village (elliptical shape). (g) and (h) The watchtower in Qiugou village (circular shape).

Their architectural characteristics can be categorized into three types according to height. Type Ⅰ, with a height below 5 meters (Fig 5a and 5b), accounts for 67.35% of the watchtowers along the ZXGW, and offered the least defensive advantage. Type Ⅱ, between 5 and 10 meters high (Fig 5c and 5d), accounts for 29.45%. Type Ⅲ, with a height of 10 meters or more, accounts for 3.21% (Fig 5e and 5f), is the tallest and bigger class of watchtowers, boasting the most prominent defensive advantage. In terms of planar shape, watchtowers can also be categorized into three types. Type A features irregular shapes (Fig 6a and 6b), accounting for 57.73%; Type B includes rectangular shapes (Fig 6c and 6d) that account for 14.58%; Type C incorporates circular or elliptical watchtowers (Fig 6e, 6f, 6g, and 6h), accounting for 27.70%.

**Fig 7:**
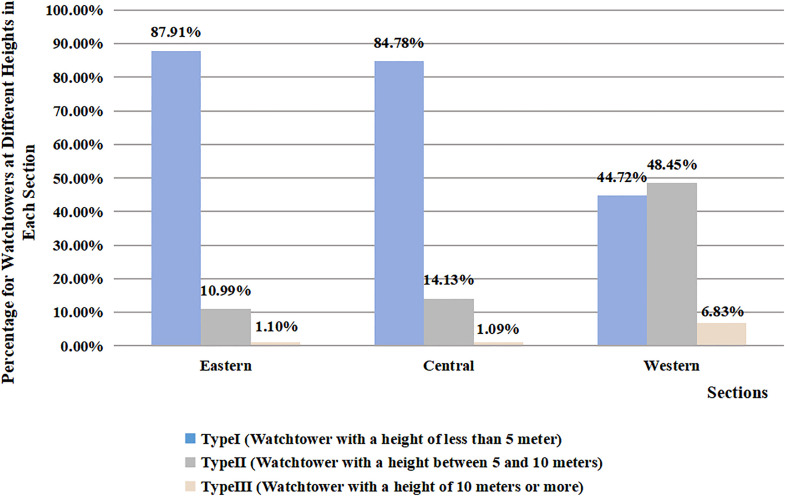
Proportional distribution of watchtower’ heights along the ZXGW in Shaanxi province across eastern, central, and western sectors. The horizontal coordinates represent distinct regions, while the vertical coordinates signify the distribution of watchtowers based on their heights across eastern, central, and western regions. The hues employed are as follows: light blue denotes watchtowers of type I, characterized by a height of less than 5 meters; light gray represents watchtowers of type II, with heights ranging from 5 to 10 meters; and flesh pink is used for watchtowers of type III, which exceed 10 meters in height.

According to statistics and typology, significant differences can be observed in the height of watchtowers along each section of the Great Wall (Fig 7). Type Ⅰ maintains an absolute numerical advantage in the eastern and middle sections, accounting for over 80% of the watchtowers along the ZXGW. Type Ⅱ is prevalent in the western section, where it accounts for 48.45%, while Type Ⅰ only accounts for 44.72% and Type Ⅲ for 6.83%. And the linear regression analysis revealed a significant association between the height of watchtowers and section (B = 1.22 meters/region level, p < 0.001). The section factor accounted for 10.2% of the variance in watchtower height (R^2^ = 0.102) (Table 1). A higher watchtower can, on one hand, increase the elevation difference between the top of the platform and the ground, providing soldiers with improved visibility and shooting perspectives. On the other hand, it can make the platform more conspicuous and enhance its capability for information transmission. So it’s clear that, the watchtowers in the western section possess stronger defensive capabilities compared to those in the eastern and middle sections, and this defensive advantage is further accentuated by the geographical environment of the western region, as the section extends across the highest elevation and the most undulating terrain along the Great Wall, with the Baiyu Mountain Range on the north side forming an additional, natural defensive barrier.

**Fig 8:**
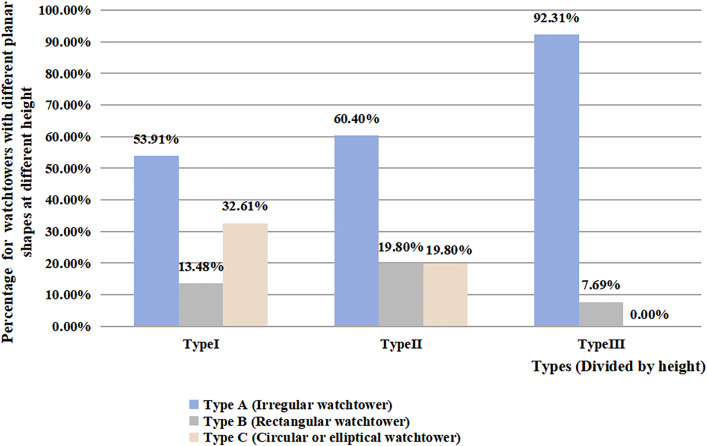
Proportionate distribution of watchtowers in plan form at different heights along the ZXGW in Shaanxi province. The horizontal coordinates in the figure represent watchtowers of different heights, and the vertical coordinates represent the percentage of watchtowers’ planforms under different height zones. Light blue indicates the watchtower of type A, that is, the plane shape is irregular; light gray indicates the watchtower of type B, that is, the plane shape is square; flesh pink indicates the watchtower of type C that is, the plane shape is round or oval.

Watchtowers can also be classified by shape as type A (irregular shape), type B (rectangular shape), and type C (round or oval). There is a certain correlation between the shape of watchtowers and their height, but the strength of the correlation is very low (B = −0.58, p = 0.003, R^2^ = 0.025) (Table 1). Among the watchtowers of different heights, type A is the most numerous. In addition, in type I, there are significantly more C-type than B-type watchtowers. In Type II, the proportion of Type B and Type C is almost balanced, whereas Type III includes no Type C watchtowers (Fig 8). This suggests that builders tended to choose round or oval designs for the lower watchtowers and rectangular ones for the higher watchtowers, though there were no strict regulations on the shape selection for watchtowers of different heights.

## 4. The distribution of watchtowers

### 4.1. Overall distribution characteristics

The watchtowers along the ZXGW are positioned in a northeast-southwest direction on the northern Shaanxi Plateau, creating a territorial boundary with other Great Wall facilities on the northwest side of the Qin state. Influenced by the historical background and natural environment, the geomorphological environment of the inner and outer sections in different parts of the ZXGW is inconsistent, and many sections are distributed along rivers.

**Fig 9:**
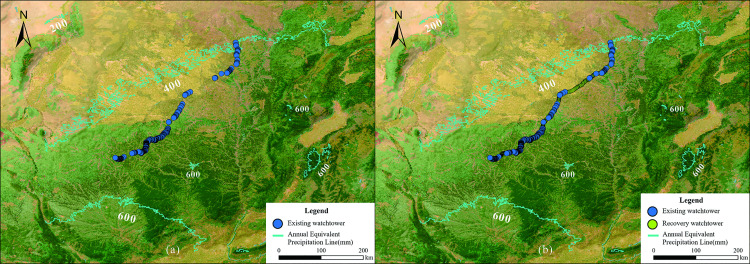
Distribution of watchtowers along the ZXGW in Shaanxi Province before and after recovery and annual precipitation. The base data was from Esri, Vizzuality, and the Half-Earth Project: https://nation.maps.arcgis.com/apps/mapviewer/index.html?webmap=d27a3805595e4bb598678f2dc3b1fca3. The blue dots in the figure represent the existing watchtowers; the yellow dots represent the watchtowers after they have been recovery in accordance with the restoration spacing of the different areas. The cyan line shows the annual precipitation.

In the eastern section, the watchtowers are generally distributed along the 400 mm equivalent precipitation line and the 3° terrain slope of Shaanxi (Fig 9), which functions as the dividing line between the flat and vast grassland pastoral area and the gully-crossed Loess Plateau agricultural area [61]. In contrast, the central and western sections of the Great Wall are positioned further south, leaving some agricultural areas outside the Great Wall.

The arrangement of the watchtowers along the ZXGW resembles a river dam intercepting the waters at their source in the northern Shaanxi Plateau and diverting them towards the area within the wall (Fig 1). This choice is indicative of the Qin people’s ingenuity to consider terrain variations when positioning the watchtowers. The river valley was the route of choice for invaders to attack the Qin state owing to convenient access to water and relatively flat roads, hence the ability to intercept enemy forces precisely at the entrance to the river valley was tantamount to the planning of defensive fortifications. Simultaneously, this positioning ensured military control of water supplies for the buildings and forts along the wall, as well as for areas in the interior.

### 4.2. Topography and density

The preserved watchtowers along the ZXGW in Shaanxi are unevenly distributed and comprise two major areas of sparse and dense distribution closely integrated with the terrain. In the Baiyu Mountain Range of the western and middle section, the watchtowers are relatively dense, while in the plateau of the eastern section, the watchtowers are relatively sparse. And linear regression analysis revealed a strong association between watchtower density and their respective sections (B = 507.43 units/level, p < 0.001, R^2^ = 0.60) (Table 1), with the density in the western section being significantly higher than that in the eastern section. In the eastern section, there is an area appears almost blank on the density map and the watchtower density is extremely low. This may be related to the local distribution of rivers. The Wuding River, the largest river in northern Shaanxi that traverses the blank area, together with its main tributary, the Yuxi River, intersect this area in a northwest-southeast and northeast-southwest direction to form a natural defense barrier and deter the southward migration of nomadic tribes. Therefore, this area does not significantly differ from surrounding areas in defense requirements and does not need to be discussed as a separate zone.

**Fig 10:**
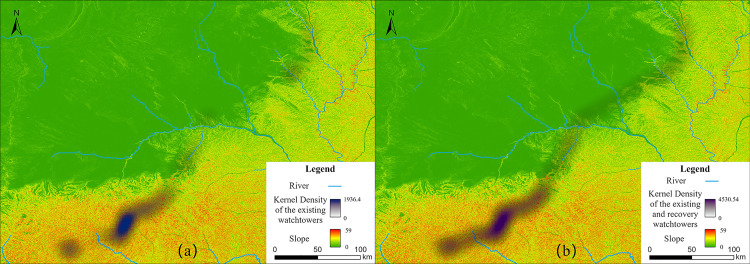
Comparison of existing and recovery kernel densities of watchtowers along the ZXGW in Shaanxi province. The base data was from USGS Earth Explorer: https://earthexplorer.usgs.gov/. **(a)** Existing Kernel Densities of Watchtowers. **(b)** Recovery Kernel Densities of Watchtowers. The darker shading in the figure represents the density of the watchtower distribution, with darker shades indicating higher densities. The colors red, yellow, and green denote the slope of the watchtower area, where red signifies a steep slope, green indicates a gentle slope, and yellow represents an average slope.

There are two possibilities for the formation of the distribution of watchtowers: firstly, the watchtowers in the Warring States period were purposefully built in this way; secondly, the sparseness of the watchtowers was once different from that of the condition at present, after suffering natural and human-made damage unevenly. In order to rule out the second possibility, this article calculates the Recovery Density of the watchtowers (Recovery Density = number of remaining watchtowers/ length of remaining wall). The quantitative examination of the watchtowers along Shaanxi’s ZXGW reveals a clear spatial pattern in the distribution density. Currently preserved structures indicate a west-east density gradient with 16,13 and 3 watchtowers per kilometer respectively in western, central and eastern section. The reconstructions of the original Warring States-period configuration reveal an even more pronounced gradient, featuring densities of 42, 33 and 13watchtowers per kilometer in western, central and eastern section. Importantly, a comparative analysis between these ancient and modern distributions underscores the enduring nature of this spatial pattern across different temporal scales. This consistently demonstrates the highest defensive concentration in the western sector—with densities 3.2 to 5.2 times greater than the eastern sector—an intermediate density in the central sector, and the least dispersion in the eastern sector (Fig 10).

The watchtowers are most densely distributed in the western section, slightly sparser in the middle section, and more than three times sparser in the eastern section than in the western section. That is, the comparison of the distribution density of watchtowers in the eastern, central, and western regions shows that, the eastern section, a low-altitude hilly area but with the sparsest watchtower distribution, was not a key defensive area for the rulers. In contrast, the central and western regions traverse higher terrain and steeper slopes, yet comprise a much higher distribution density of watchtowers than the eastern region. Obviously, they were areas wherein rulers deliberately increased their fortification efforts.

### 4.3. Spatial distribution

**Fig 11:**
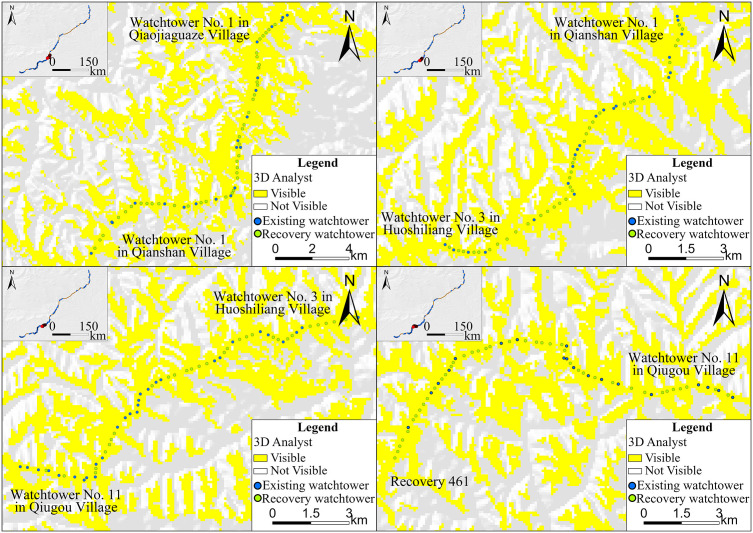
Viewshed analysis of existing and recovery watchtowers in the central section of the ZXGW in Shaanxi province. The base data was from: USGS Earth Explorer: https://earthexplorer.usgs.gov/. Blue dots represent existing watchtowers, while green dots denote points recovery ones based on the restoration spacing. Mutually visible areas are highlighted in yellow, and non-visible areas in white. (a)Watchtower No. 1 in Qiaojiaguaze village—Watchtower No. 1 in Qianshan village(from east to west) (b)Watchtower No. 1 in Qianshan village—Watchtower No. 3 in Huoshiliang village(from east to west) (c)Watchtower No. 3 in Huoshiliang village—Watchtower No. 11 in Qiugou village(from east to west) (d)Watchtower No. 11 in Qiugou village—Recovery 461(from east to west).

**Fig 12:**
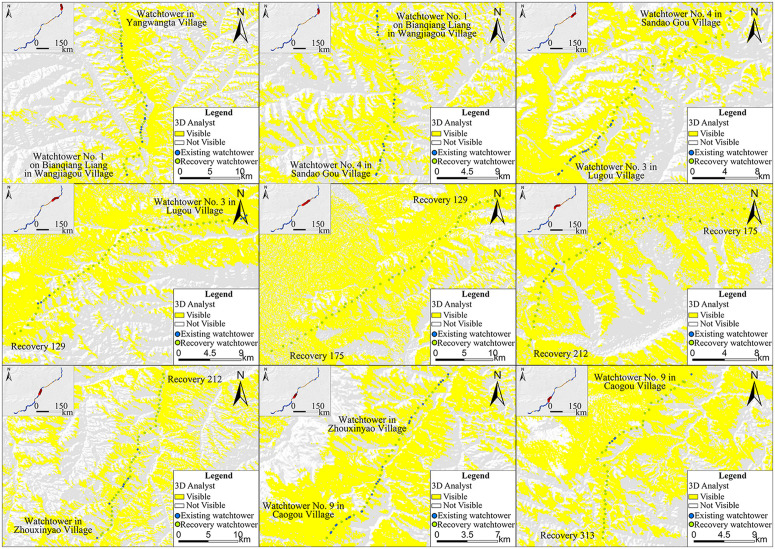
Viewshed analysis of existing and recovery watchtowers in the eastern section of the ZXGWV in Shaanxi Province. The base data was from: USGS Earth Explorer: https://earthexplorer.usgs.gov/. Blue dots represent existing watchtowers, while green dots denote points recovery ones based on the restoration spacing. Mutually visible areas are highlighted in yellow, and non-visible areas in white. (a)Watchtower in Yangwangta village—Watchtower No. 1 on Bianqiang Liang in Wangjiagou village(from east to west) (b)Watchtower No. 1 on Bianqiang Liang in Wangjiagou village—Watchtower No. 4 in Sandao Gou village(from east to west) (c)Watchtower No. 4 in Sandao Gou village—Watchtower No. 3 in Lugou village(from east to west) (d)Watchtower No. 3 in Lugou village—Recovery 129(from east to west) (e)Recovery 129—Recovery 175(from east to west) (f)Recovery 175—Recovery 212(from east to west) (g)Recovery 212—Watchtower in Zhouxinyao village(from east to west) (h)Watchtower in Zhouxinyao village—Watchtower No. 9 in Caogou village(from east to west) (i)Watchtower No. 9 in Caogou village—Recovery 313(from east to west).

The shape and killing range of bows and crossbows differed across Chinese history. Sun Bin’s *Art of War*, authored in the Warring States period – the same time the ZXGW was built – records that the Qin crossbow could kill at a distance of 100 paces, which equals to a little over 140 meters [62]. Archaeological findings and restorations confirm this attestation. For example, restorations of a large number of crossbows unearthed in the mausoleum of the First Emperor of Qin suggest a range of about 300 meters for the Qin crossbow and an effective killing distance of about 150 meters [2].The recovery spacing of Shaanxi province’s western section of the ZXGW is 240.01 meters, the lowest among the three sections. That is to say, if an invading force appeared in the western section, regardless of its position, the watchtowers would have allowed the Qin army to engage with the enemy at an effective range. The watchtowers in the central section, with the recovery spacing of 299.13 meters. Although most of the watchtowers in this area are not sufficient to meet the requirements for weapon reach, they still followed certain principles. Upon conducting a comprehensive visual field analysis of all watchtowers situated in the central sector, the findings indicate that the most are within the visual range of two proximate watchtowers. Importantly, not only are the recovery ones mutually visible, but adjacent watchtowers—even those are only the existing ones—are also within each other’s field of vision (Fig 11). Hence, the positioning of these watchtowers may have been dictated by the requirement for mutual visibility, which means the builders tried to ensure that the watchtowers were visible to adjacent ones. Although compared with the ‌principle of weapon reach, it is a compromise to the huge loss of manpower and material resources brought by the construction, the watchtowers can still form unobstructed information transmission channels between watchtowers and between watchtowers and other subsidiary facilities of the Great Wall. The recovery spacing of 749.61 meters between the watchtowers in the eastern section is the widest among the three sections and clearly exceeds the effective range of the Qin crossbow. As far as the area of vision between the watchtowers is concerned,the analysis on ArcGIS indicates that some of the neighboring watchtowers in the east section are visible to each other but many are not, even with the consideration of the recovery ones (Fig 12). As a result, neither the effective range of missile weapons nor the field of vision were major considerations in the construction of the watchtowers in the eastern sector, which contributed little to the defensive advantage and whose significance may have been symbolic, that is, to draw a distinct line between “our side” and “the other side”.

### 4.4. Comparison of the defensive advantages of watchtowers in each section

The defensive functions and advantages of the watchtowers along the ZXGW vary across different sections. Those in the western section offer the most prominent defensive advantages: situated on the southern side of Baiyu Mountain, they benefited from a natural defensive formation across the steep northern mountains. In addition, the watchtowers in the section are the tallest ones (most of them over 5 meters) and the most densely distributed: with an average recovery spacing of 240.01 meters, they allowed for full coverage of missile weapon range along the Great Wall. The watchtowers in the middle section are positioned on the northern side of Baiyu Mountain and exhibit transition characteristics in defensive compared with the other two sections. The elevated and undulating terrain provided a more substantial defensive advantage than the eastern section. However, compared to the positioning of the watchtowers and the Baiyu Mountain in the western section, the utilization of mountains by the watchtowers in the middle section is slightly inferior. The distribution of watchtowers in this section (recovery average spacing of 299.13 meters) is more dense than the watchtowers in the eastern section and less dense than that in the western section. Spacing abandoned firing range but ensured mutual visibility between neighboring watchtowers. Located on a flat plateau with low terrain, the watchtowers in the eastern section provide the least defensive advantage among the three sections of the ZXGW in Shaanxi Province. Moreover, they are more sparsely spaced, with a recovery spacing of 749.61 meters. Despite low elevation and wide sight areas from the top of watchtowers, many neighboring watchtowers were not mutually visible and hard to make it play a strategic role as a defense system, hence their value lay more in the symbolic rather than the practical realm (Table 3).

**Table 3:** Analysis of the Defense Capabilities of Watchtowers in Different Regions along the ZXGW in Shaanxi Province.

Section			
**Characteristics**	**Eastern Section**	**middle section**	**Western Section**
**Topography**	Low elevation and mild terrain fluctuations.	High elevation with significant terrain fluctuations.	High elevation with significant terrain fluctuations and the northern high mountain
**Watchtower Height**	Mainly less than 5 meters.		Mainly over 5 meters.
**Distribution Density**	Relatively sparse	Medium	Relatively dense
**Average recovery spacing (m)**	749.61	299.13	240.01
**Spacing Layout Principle**	Demarcation of national boundaries.	Mutual visibility	A continuous area within ranged weapon reach
**Defensive Advantage**	Low	Medium	High

Historical conditions at the beginning of the erection of these buildings had played a role. These watchtowers were built during the Warring States period, when the Qin state intended to stabilize its western flank and expand against all six states in the east. However, with a length of about 462 kilometers, the Great Wall traversed different areas with significantly different defensive requirements. The protection of the newly acquired territory in Yiqu on the southern side of the western section of the ZXGW in Shaanxi Province [63] was highly likely to be the main reason the Qin state built extensive fortifications in this area. Therefore, the western section is a key defensive area for the Great Wall, and the defensive advantages of this section are also the most significant. The eastern section borders the highly mobile nomadic tribes to the northwest, and all had submitted to Qin [56]. Thus, watchtowers therein provided less significant defensive advantages compared to those in the western section. Finally, watchtowers in the middle section occupy the middle ground between the other two sections in terms of defensive advantages.

## 5. Conclusion

This research conducts an analysis of 347 watchtowers situated along Shaanxi’s ZXGW line, with the aim of elucidating the adaptive construction principles employed by the Qin State during the Warring States period. The key findings are as follows:

The architectural design of watchtowers comprised rammed earth platforms that protruded from the wall, and were either rectangular or elliptical/circular in shape. The watchtowers in the western section, which were between 5-10m in height, were primarily rectangular, while the watchtowers in the eastern section, which were less than 5m in height, were predominantly elliptical. This suggests the presence of regional variations in construction strategies.

As for the distribution Patterns, the density of watchtowers displays a west-to-east gradient, with the highest density in the west, followed by the middle, and the lowest in the east. In the west, watchtowers were strategically placed within the double Qin crossbow range to ensure overlapping defense coverage. In contrast, the watchtowers in the middle were primarily designed for intervisibility, emphasizing communication. The sparse distribution of watchtowers in the east suggests their symbolic significance rather than a defensive one.

From a strategic standpoint, the formidable defensive structures in the western sector appear to correlate with the frequency of wars and newly subjugated Yiqu territories. Conversely, the eastern watchtowers suggest a moderate level of military pressure. This vividly illustrates the Qin’s adept strategic response to local threats and topographical conditions.

## Supporting information

S1 Table Information on existing watchtowers along the ZXGW in Shaanxi Province. The individual dimensions of the watchtowers presented in the table remain unmeasurable due to their subsequent collapse.
(XLSX)

S2 Table Information on the starting,ending and route points of the ZXGW in Shaanxi Province. (XLSX)

S3 Table Information on recovery watchtowers along the ZXGW in Shaanxi Province. (XLSX)

S4 Table Survey registration form for watchtowers along the ZXGW in Shaanxi province. Take Tuantuangou village No.2 watchtower as the example.
(PDF)

S1 Fig Pictures of the well-preserved watchtowers in the eastern section. (a) Watchtower No. 2 in Tuantuangou village. (b) Watchtower No. 5 in Tuantuan Gou village. (c) Watchtower No. 1 in Nangeila village. (d) Watchtower No. 3 in Nangeila village. (e)Watchtower No. 2 in Lamagou village. (f) Watchtower in Dujiayaozi village. (g) Watchtower No. 1 in Dongzuojie Villag. (h) Watchtower in Xiaojiamao village. (i) Watchtower No. 1 in Guchengjie village. (j) Watchtower No. 3 in Guchengjie village. (k) Watchtower No. 5 in Guchengjie village. (l) Watchtower in Kangliang village. (m) Watchtower in Shawozhuang village. (n) Watchtower in Chengshan Villag. (o) Watchtower No. 1 in Shimiaogou village. (p) Watchtower No. 2 in Shimiaogou village. (q) Watchtower No. 2 in Xiangshuitang village. (r) Watchtower No. 2 in Lugou village. (s) Watchtower in Yanqucha village. (t) Watchtower No. 1 in Qinghegou village.(u) Watchtower No. 2 in Qinghegou village.(v) Watchtower No. 3 in Qinghegou village.(w) Watchtower No. 8 in Caogou village. (a)-(w)were provided by Xingyi Li.
(PDF)

S2 Fig Pictures of the well-preserved watchtowers in the central section. (a) Watchtower No. 1 in Yinwan village. (b) Watchtower No. 2 in Yinwan village. (c) Watchtower in Liujian village. (d) Watchtower No. 1 in Qianshan village. (e) Watchtower No. 4 in Ningtiaowan village. (f) Watchtower No. 1 in Niandaowan village. (g) Watchtower No. 7 in Chaijiawan village. (h) Watchtower No. 3 in Qiugou village. (i) Watchtower No. 5 in Qiugou village. (j) Watchtower No. 12 in Qiugou village. (k) Watchtower No. 13 in Qiugou village. (l) Watchtower No. 1 in Yushutai village. (m) Watchtower No. 2 in Yushutai village. (n) Watchtower No. 4 in Yushutai village. (o) Watchtower No. 3 in Heilonggou village. (a)-(o)were provided by Xingyi Li.
(PDF)

S3 Fig Pictures of the well-preserved watchtowers in the western section. (a) Watchtower No. 3 in Yangjiagou village.(b) Watchtower No. 8 in Yangxinzhuang village.(c) Watchtower No. 16 in Yangxinzhuang village.(d) Watchtower No. 4 in Shuangmiao village.(e) Watchtower No. 8 in Shuangmiao village.(f) Watchtower No. 1 in Liugou village.(g) Watchtower No. 1 in Heyangwan village.(h) Watchtower No. 3 in Heyangwan village.(i) Watchtower in Liubian village. (j) Watchtower No. 2 in Zhongyangqing village. (k) Watchtower No. 2 in Yangwa village. (l) Watchtower No. 1 in Dongjian village.(m) Watchtower No. 2 in Xijian village. (n) Watchtower No. 1 in Lingouliang village. (o) Watchtower No. 2 in Liushuyaoxian village. (p) Watchtower No. 3 in Yayaowan village. (q) Watchtower No. 2 in Suancigou village. (r) Watchtower No. 3 in Huangcaowa village. (s) Watchtower No. 6 in Huangcaowa village. (t) Watchtower No. 3 in Liuzhuang village. (u) Watchtower No. 1 in Malinyaoxian village. (v) Watchtower No. 2 in Malinyaoxian village. (w) Watchtower No. 3 in Malinyaoxian village. (x) Watchtower No. 5 in Malinyaoxian village. (y) Watchtower No. 6 in Malinyaoxian village. (a)-(y)were provided by Xingyi Li.
(PDF)
