# Supplementary material for: The building concept of border defence facilities of Qin: Watchtowers along the King of Zhaoxiang great wall in Shaanxi province
Source: PLoS One. 2025 Aug 26;20(8):e0329298. doi: 10.1371/journal.pone.0329298 (PMC12380348; doi:10.1371/journal.pone.0329298)
Supplement: S4 Table — Take Tuantuangou village No.2 watchtower as the example. (PDF) [file pone.0329298.s004.pdf]

Survey Registration Form for Watchtowers along the ZXGW in Shaanxi Province

(Take Tuantuangou village No.2 watchtower as the example)

County**: Shenmu County, Yulin City, Shaanxi Province**

<table>
<tr><td>Name</td><td colspan="2">Tuantuangou Village No.2 Watchtower</td><td>Other Names</td><td>None</td></tr>
<tr><td rowspan="2">Location</td><td>Site</td><td colspan="3">East of Tuantuangou Village, Gaojiabao Town</td></tr>
<tr><td>Coordinates</td><td colspan="3">East Longitude: 110°16′14.62″ North Latitude: 38°39′08.86″
Elevation: 1219.1 meters</td></tr>
<tr><td>Material</td><td colspan="4">□Wood □Brick □Stone ☑Earth □Other</td></tr>
<tr><td>Plan Form</td><td colspan="4">□Rectangular □Circular ☑Other</td></tr>
<tr><td>Section Form</td><td colspan="4">□Trapezoidal □Rectangular ☑Other</td></tr>
<tr><td>Preservation Status</td><td colspan="4">Current Condition: The watchtower is poorly preserved. The south, north, and west sides are upright, with exposed rammed earth layers.
Damage Details:
Damage Description: The east side has collapsed into a gentle slope. There is a "U"-shaped notch on the northeast side. The north side has a large "V"-shaped notch in the middle. The south side has collapsed into a gentle slope at the bottom. The top of the watchtower is irregularly collapsed.
Causes of Damage:
Natural Factors: Erosion from wind and rain, plant growth, and ant hole damage.
Human Factors: Local farming activities and resident encroachment.
Diseases Present: Plant growth and rainwash.</td></tr>
<tr><td>Detailed Description</td><td colspan="4">Construction Form:
The watchtower is located on Section 1 of the Great Wall in Tuantuangou Village, Shenmu County. Both the plan and section forms are irregular.
Construction Materials:
The watchtower is built with rammed earth, mixed with tiles and some stone flakes. The rammed earth layers are 0.07–0.09 meters thick. Many tile fragmentsare scattered around the watchtower.
Dimensions:
Base: 10.5 meters (E-W) × 14 meters (N-S).
Top: 10.5 meters (E-W) × 13 meters (N-S).
Remaining height: 3.3 meters.
Ancillary Facilities: None.
Repair Status: Built in the Warring States Period by the Qin State, with no signs of repair.</td></tr>
</table>

<table>
<tr>
<td></td>
<td>Planimetric Relationship with Other Ruins:
185 meters southwest of Watchtower No.3 in Tuantuangou Village and 480 meters northeast of Watchtower No.1 in Tuantuangou Village.
Relics: Tile fragments and stones.</td>
</tr>
<tr>
<td>Natural Environment</td>
<td>Geology and Topography:
The watchtower is located in a loess hilly area. It is situated 1.2 kilometers east of Tuantuangou Village in Gaojiabao Town, Shenmu County, on a gentle slope. The north side is adjacent to a deep gully. There is a gully 50 meters northwest of the watchtower. The east side is next to a mountain road. The south side has cultivated farmland on the gentle slope. The surrounding soil is severely desertified.</td>
</tr>
<tr>
<td>Photos</td>
<td>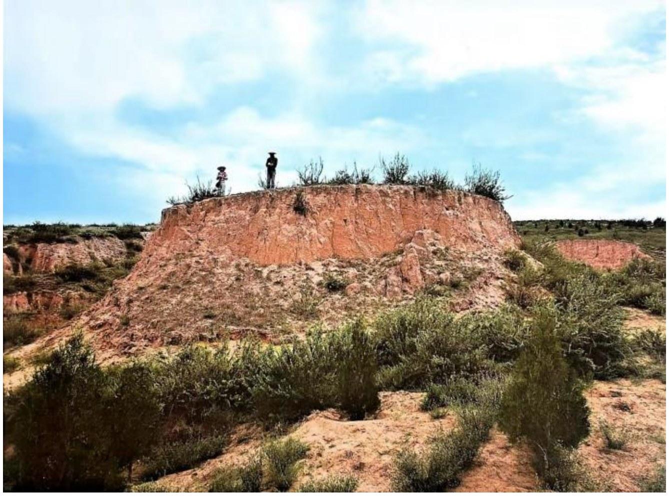
Fig.1 Tuantuangou Village No.2 Watchtower (East–West view)
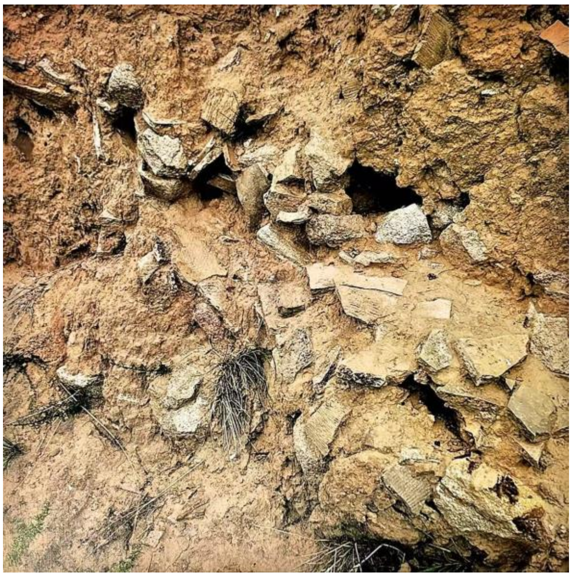
Fig.2 Tiles in the rammed earth layers of Tuantuangou Village No.2 Watchtower</td>
</tr>
</table>

**Plan and Elevation Drawings**	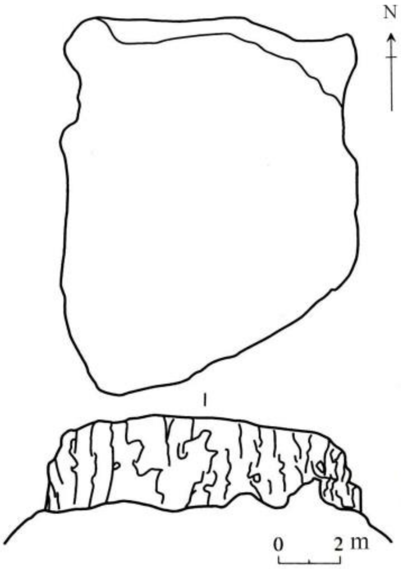<br>Fig.3 Tuantuangou Village No.2 Watchtower's Plan and elevation Drawing
