# Supplementary figures and images for: The building concept of border defence facilities of Qin: Watchtowers along the King of Zhaoxiang great wall in Shaanxi province
Source: PLoS One. 2025 Aug 26;20(8):e0329298. doi: 10.1371/journal.pone.0329298 (PMC12380348; doi:10.1371/journal.pone.0329298)

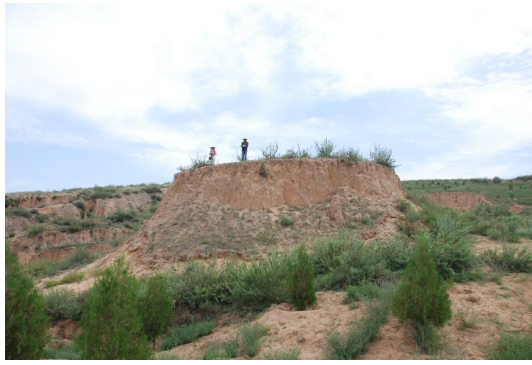

(a)

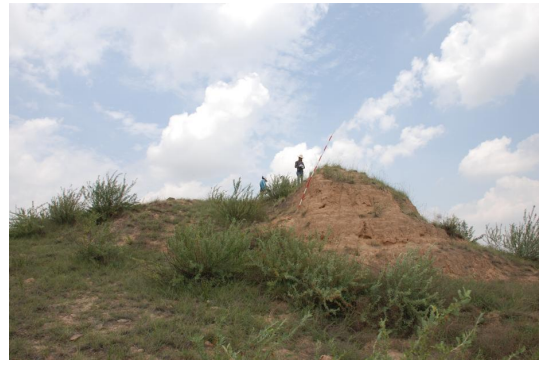

(e)

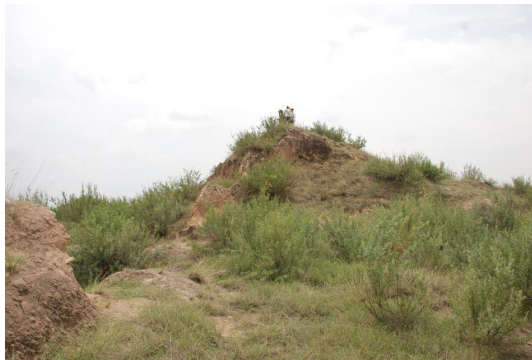

(b)

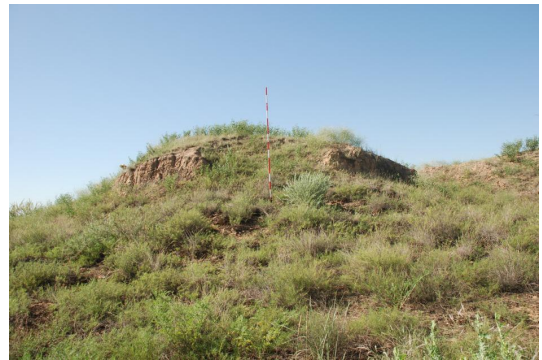

(f)

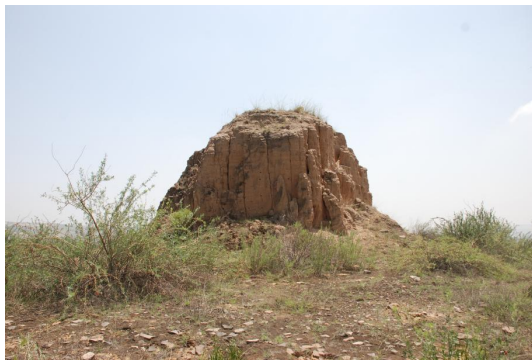

(c)

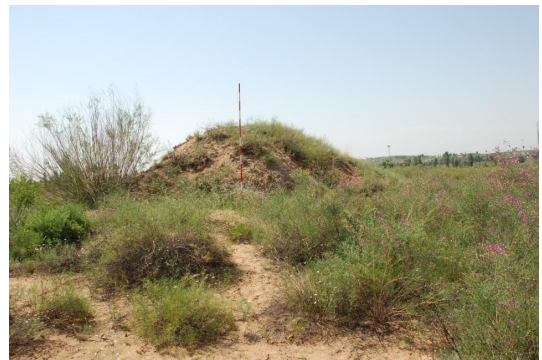

(g)

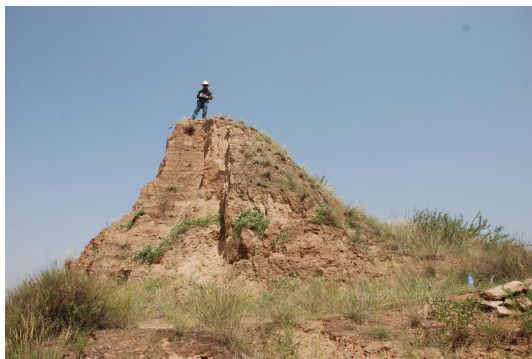

(d)

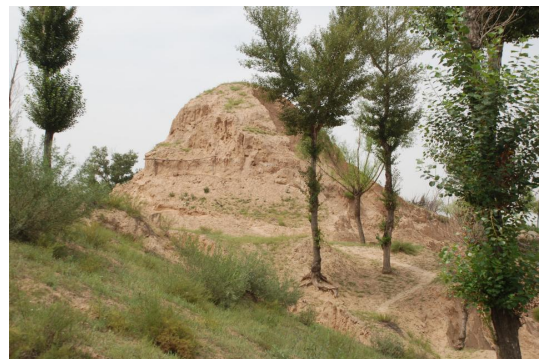

(h)

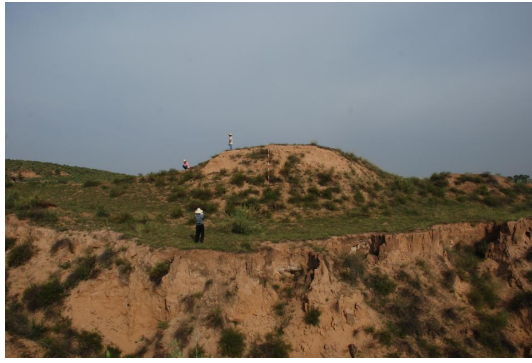

(i)

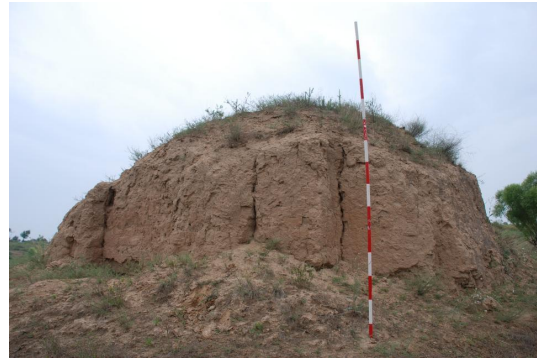

(m)

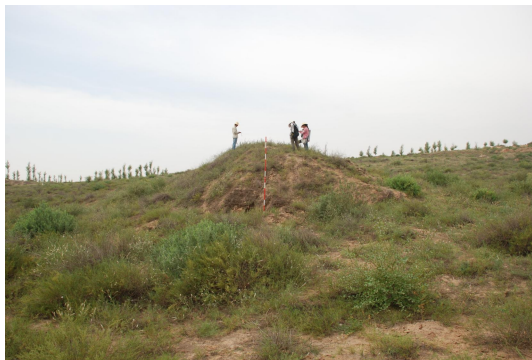

(j)

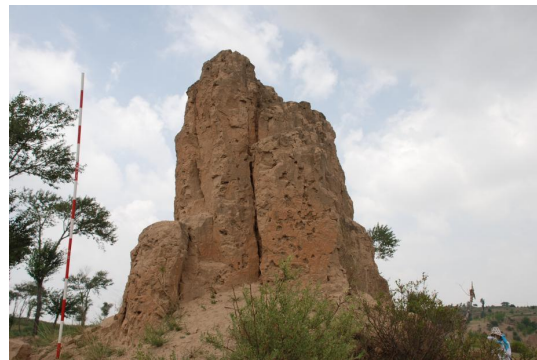

(n)

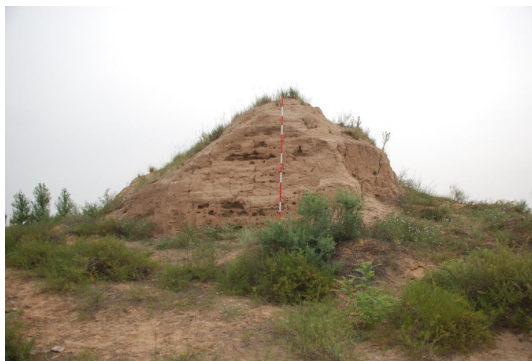

(k)

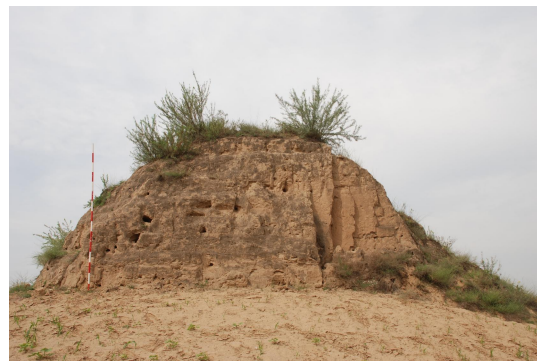

(o)

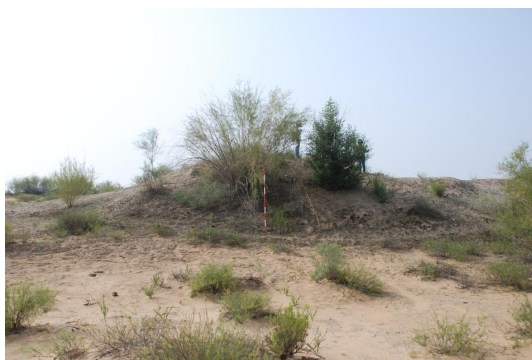

(l)

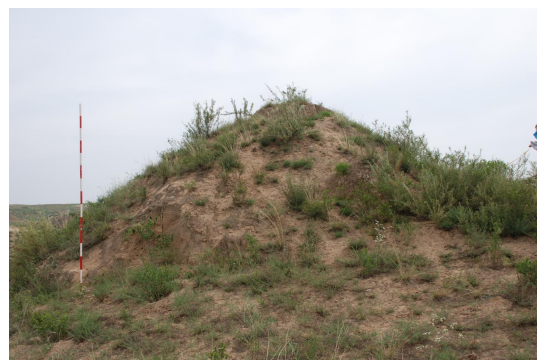

(p)

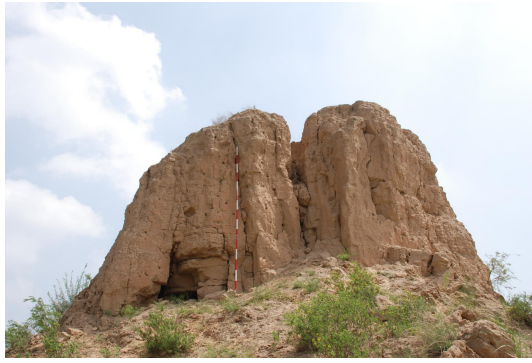

(q)

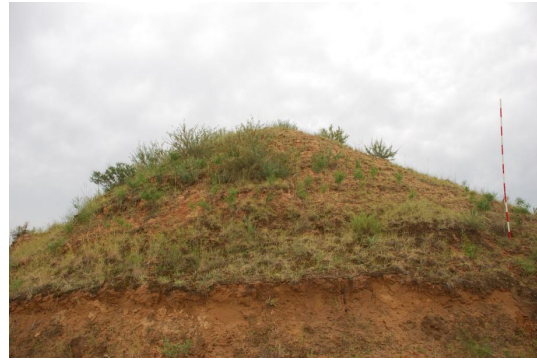

(u)

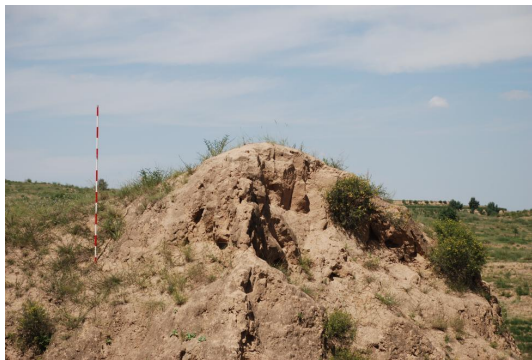

(r)

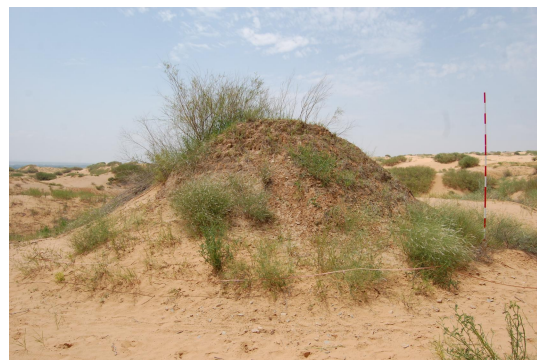

(v)

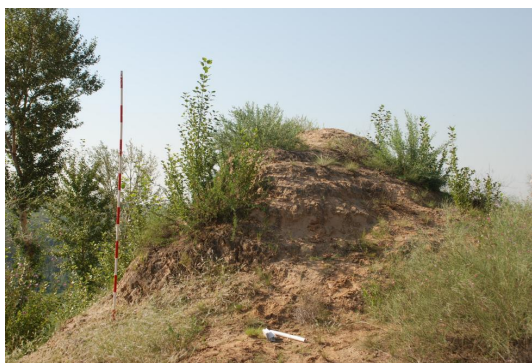

(d)

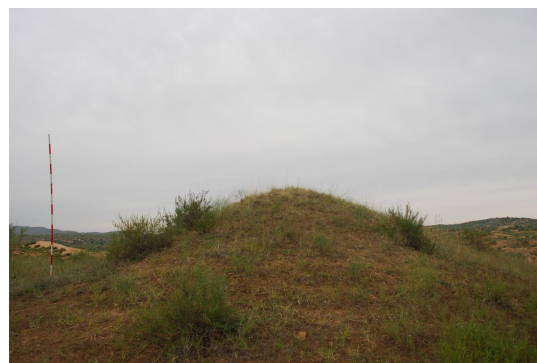

(w)

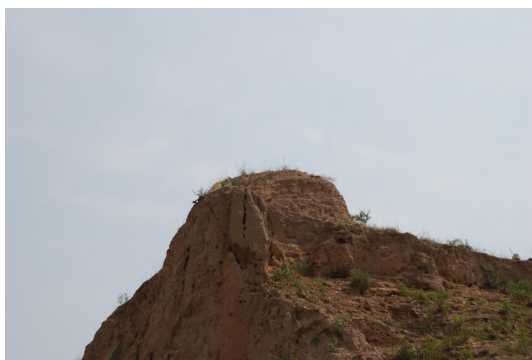

(t)

Supplement: S1 Fig — (a) Watchtower No. 2 in Tuantuangou village. (b) Watchtower No. 5 in Tuantuan Gou village. (c) Watchtower No. 1 in Nangeila village. (d) Watchtower No. 3 in Nangeila village. (e)Watchtower No. 2 in Lamagou village. (f) Watchtower in Dujiayaozi village. (g) Watchtower No. 1 in Dongzuojie Villag. (h) Watchtower in Xiaojiamao village. (i) Watchtower No. 1 in Guchengjie village. (j) Watchtower No. 3 in Guchengjie village. (k) Watchtower No. 5 in Guchengjie village. (l) Watchtower in Kangliang village. (m) Watchtower in Shawozhuang village. (n) Watchtower in Chengshan Villag. (o) Watchtower No. 1 in Shimiaogou village. (p) Watchtower No. 2 in Shimiaogou village. (q) Watchtower No. 2 in Xiangshuitang village. (r) Watchtower No. 2 in Lugou village. (s) Watchtower in Yanqucha village. (t) Watchtower No. 1 in Qinghegou village.(u) Watchtower No. 2 in Qinghegou village.(v) Watchtower No. 3 in Qinghegou village.(w) Watchtower No. 8 in Caogou village. (a)-(w)were provided by Xingyi Li. (PDF) [file pone.0329298.s005.pdf]

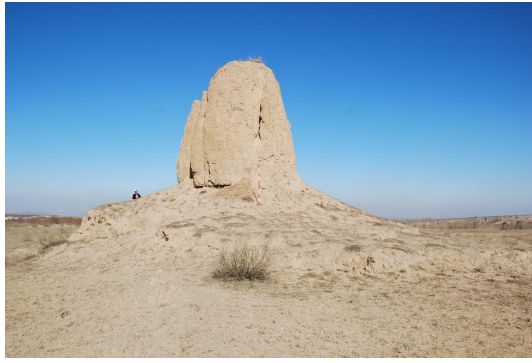

(a)

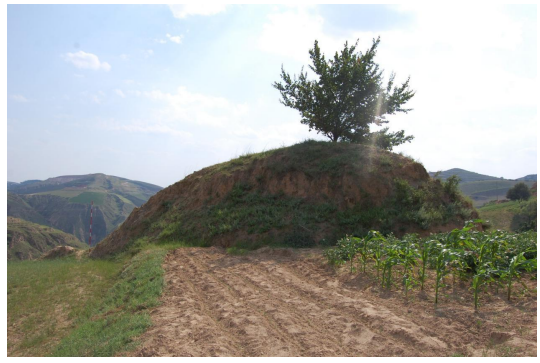

(e)

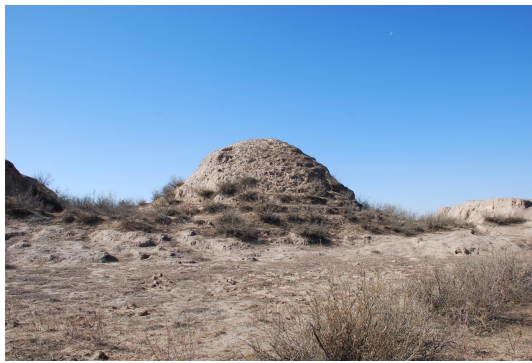

(b)

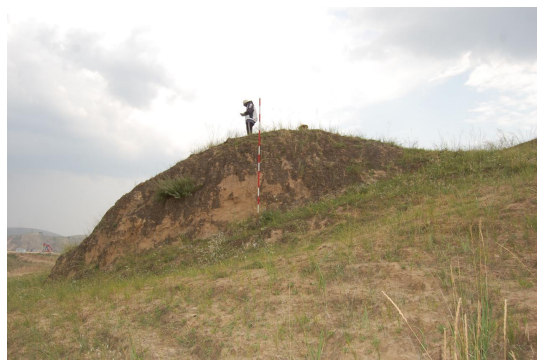

(f)

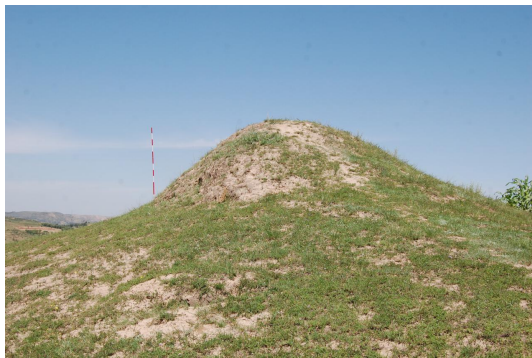

(c)

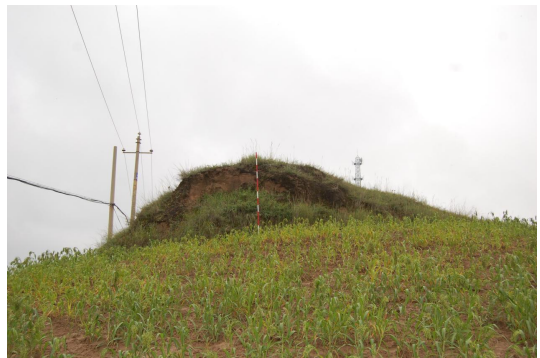

(g)

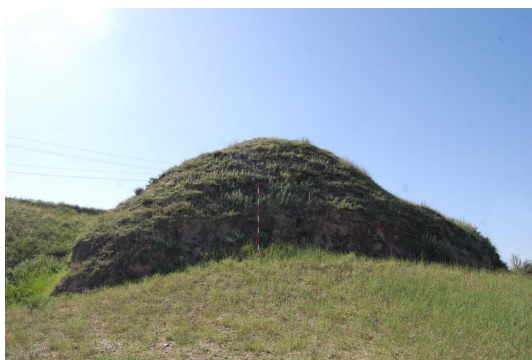

(d)

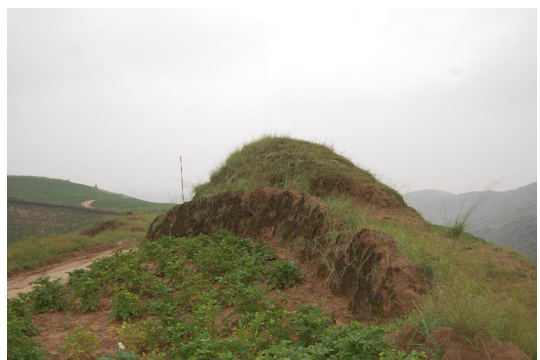

(h)

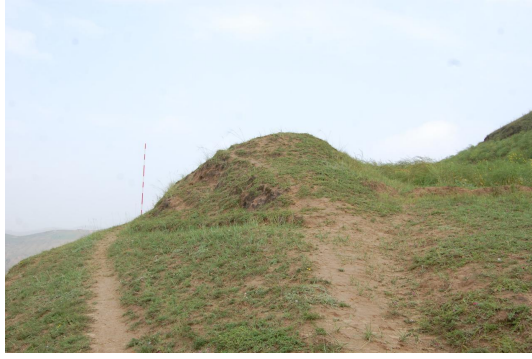

(i)

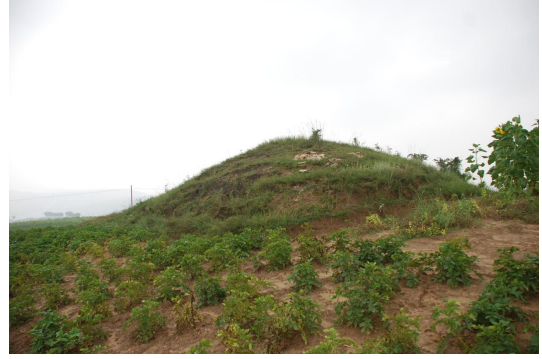

(m)

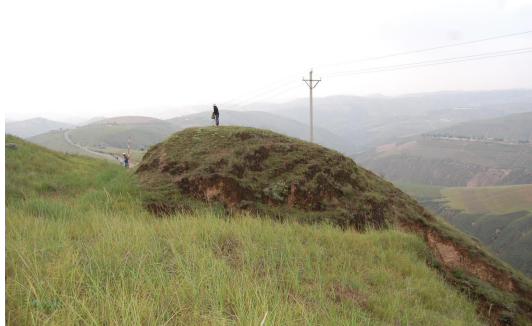

(j)

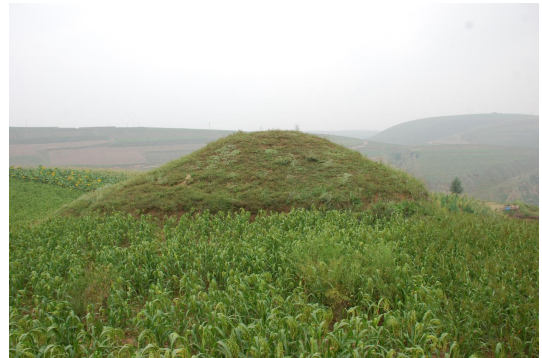

(n)

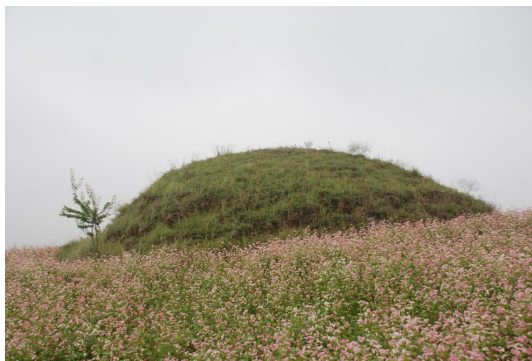

(k)

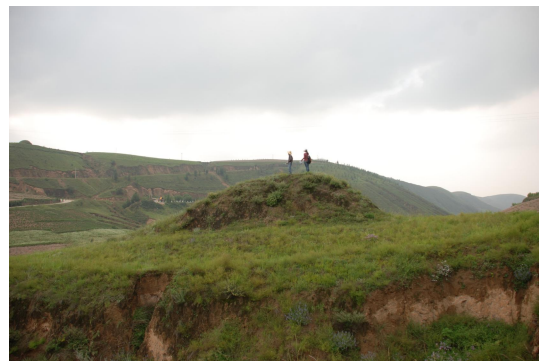

(o)

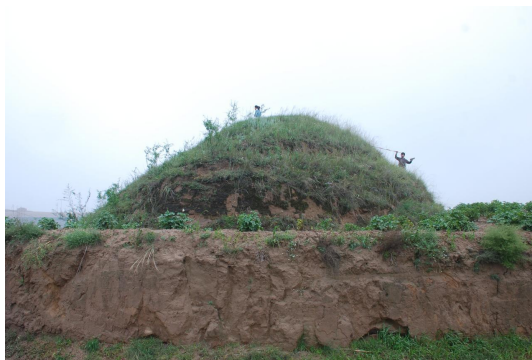

(l)

Supplement: S2 Fig — (a) Watchtower No. 1 in Yinwan village. (b) Watchtower No. 2 in Yinwan village. (c) Watchtower in Liujian village. (d) Watchtower No. 1 in Qianshan village. (e) Watchtower No. 4 in Ningtiaowan village. (f) Watchtower No. 1 in Niandaowan village. (g) Watchtower No. 7 in Chaijiawan village. (h) Watchtower No. 3 in Qiugou village. (i) Watchtower No. 5 in Qiugou village. (j) Watchtower No. 12 in Qiugou village. (k) Watchtower No. 13 in Qiugou village. (l) Watchtower No. 1 in Yushutai village. (m) Watchtower No. 2 in Yushutai village. (n) Watchtower No. 4 in Yushutai village. (o) Watchtower No. 3 in Heilonggou village. (a)-(o)were provided by Xingyi Li. (PDF) [file pone.0329298.s006.pdf]

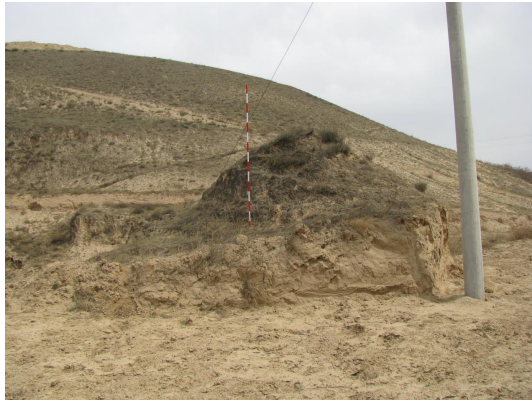

(a)

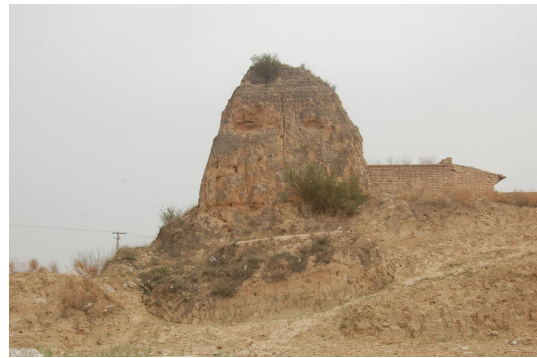

(e)

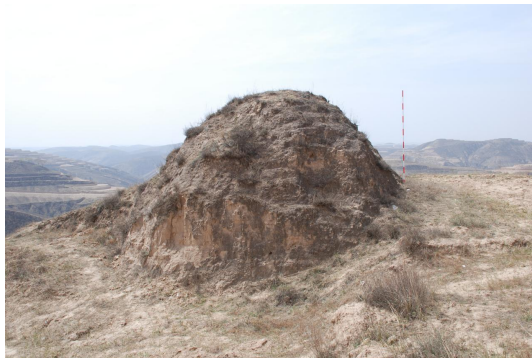

(b)

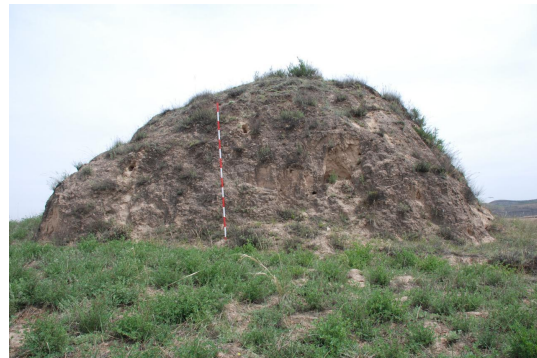

(f)

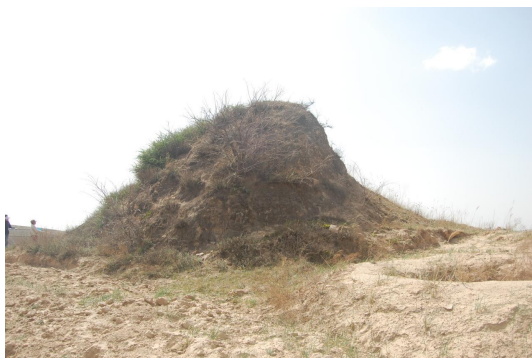

(c)

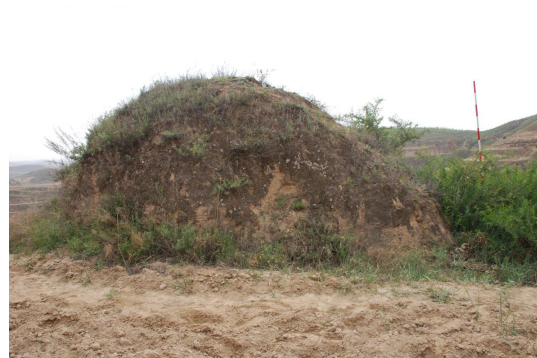

(g)

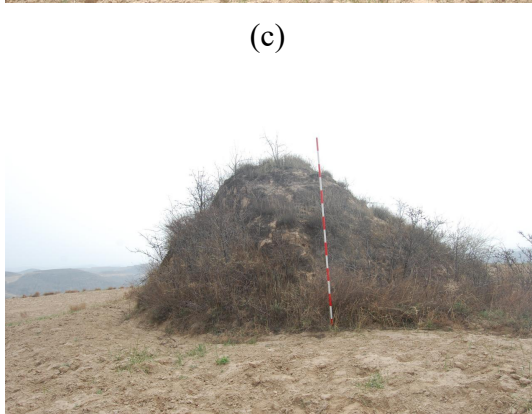

(d)

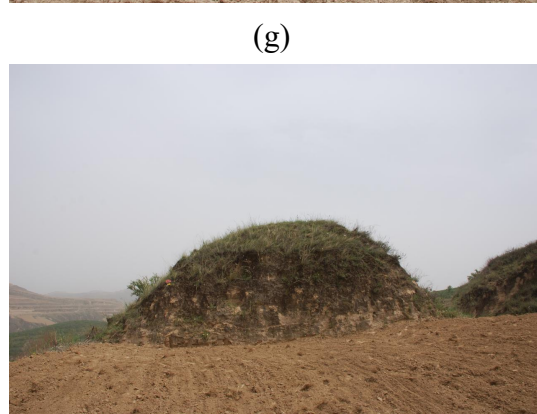

(h)

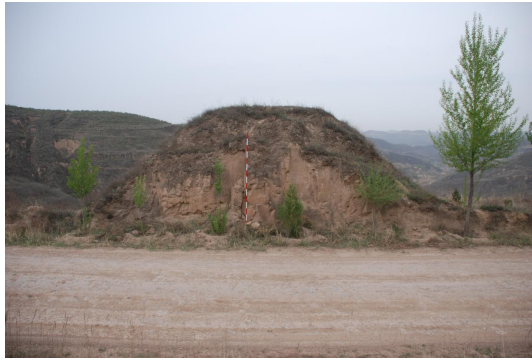

(i)

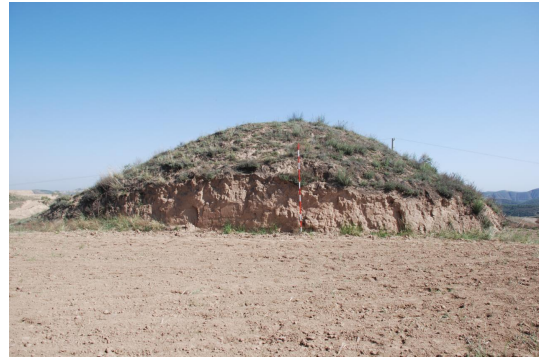

(m)

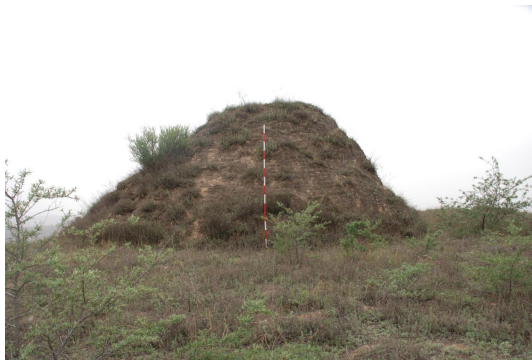

(j)

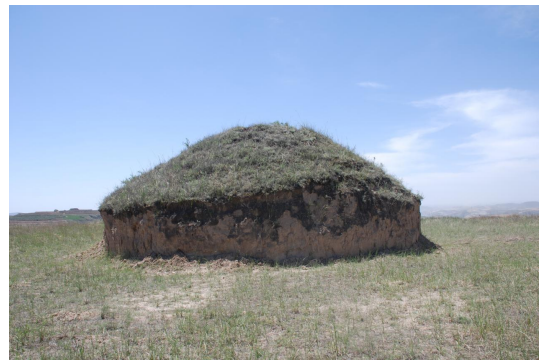

(n)

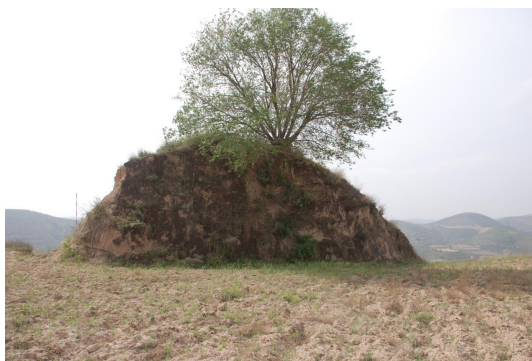

(k)

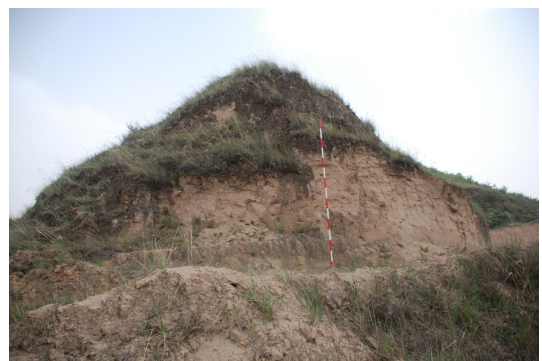

(o)

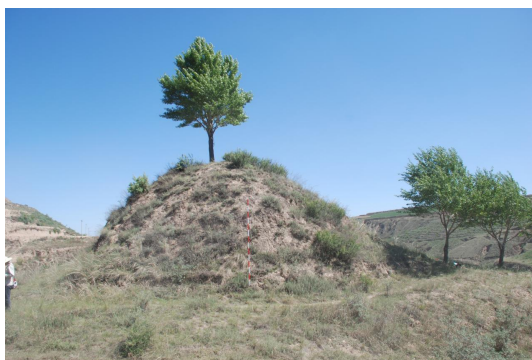

(l)

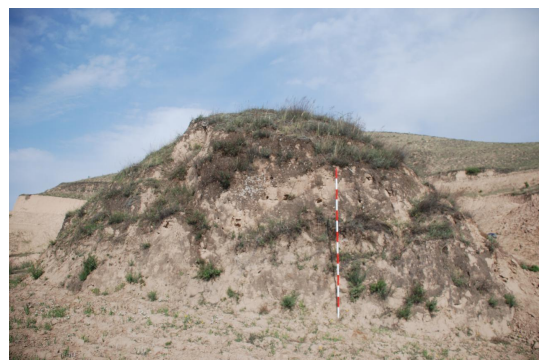

(p)

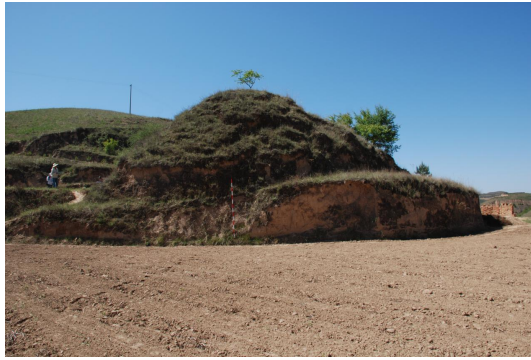

(q)

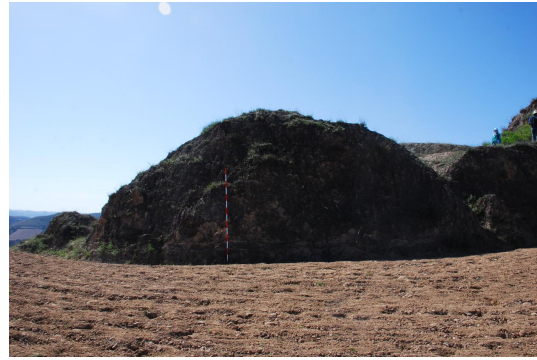

(u)

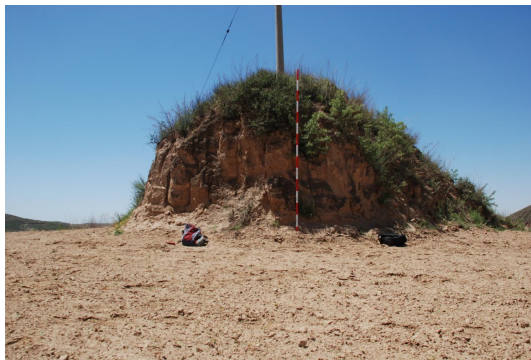

(r)

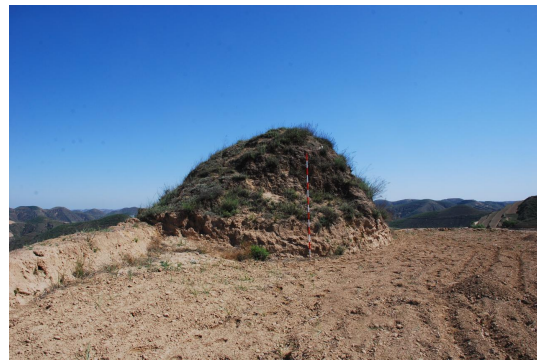

(v)

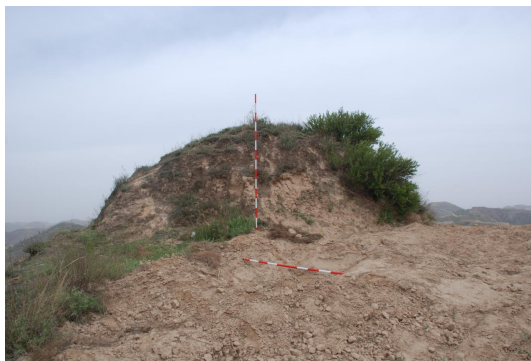

(s)

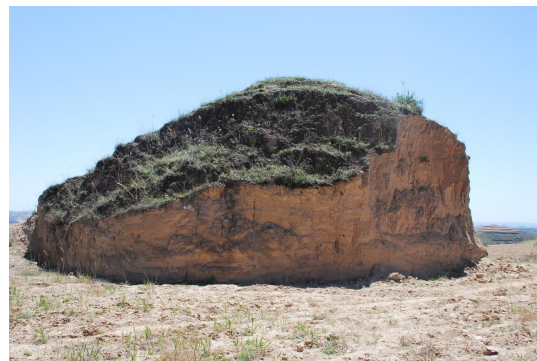

(w)

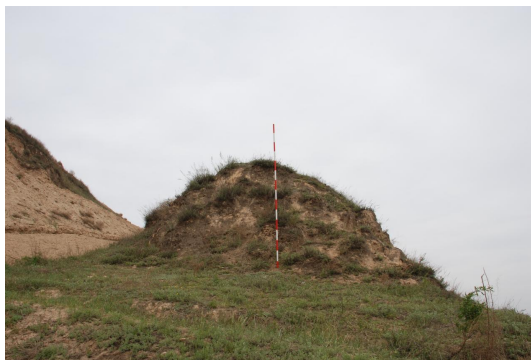

(t)

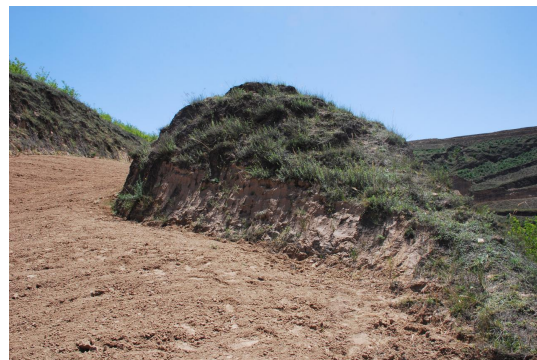

(x)

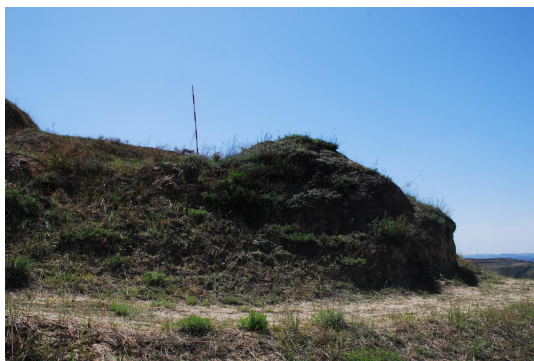

(y)

Supplement: S3 Fig — (a) Watchtower No. 3 in Yangjiagou village.(b) Watchtower No. 8 in Yangxinzhuang village.(c) Watchtower No. 16 in Yangxinzhuang village.(d) Watchtower No. 4 in Shuangmiao village.(e) Watchtower No. 8 in Shuangmiao village.(f) Watchtower No. 1 in Liugou village.(g) Watchtower No. 1 in Heyangwan village.(h) Watchtower No. 3 in Heyangwan village.(i) Watchtower in Liubian village. (j) Watchtower No. 2 in Zhongyangqing village. (k) Watchtower No. 2 in Yangwa village. (l) Watchtower No. 1 in Dongjian village.(m) Watchtower No. 2 in Xijian village. (n) Watchtower No. 1 in Lingouliang village. (o) Watchtower No. 2 in Liushuyaoxian village. (p) Watchtower No. 3 in Yayaowan village. (q) Watchtower No. 2 in Suancigou village. (r) Watchtower No. 3 in Huangcaowa village. (s) Watchtower No. 6 in Huangcaowa village. (t) Watchtower No. 3 in Liuzhuang village. (u) Watchtower No. 1 in Malinyaoxian village. (v) Watchtower No. 2 in Malinyaoxian village. (w) Watchtower No. 3 in Malinyaoxian village. (x) Watchtower No. 5 in Malinyaoxian village. (y) Watchtower No. 6 in Malinyaoxian village. (a)-(y)were provided by Xingyi Li. (PDF) [file pone.0329298.s007.pdf]
